# Mechanopathology of biofilm-like *Mycobacterium tuberculosis* cords

**DOI:** 10.1016/j.cell.2023.09.016

**Published:** 2023-11-09

**Authors:** Richa Mishra, Melanie Hannebelle, Vishal P. Patil, Anaëlle Dubois, Cristina Garcia-Mouton, Gabriela M. Kirsch, Maxime Jan, Kunal Sharma, Nicolas Guex, Jessica Sordet-Dessimoz, Jesus Perez-Gil, Manu Prakash, Graham W. Knott, Neeraj Dhar, John D. McKinney, Vivek V. Thacker

**Affiliations:** 1 Global Health Institute, École Polytechnique Fédérale de Lausanne, 1015 Lausanne, Switzerland; 2 Department of Bioengineering, Stanford University, Stanford, CA 94305, USA; 3 BioElectron Microscopy Facility, École Polytechnique Fédérale de Lausanne, 1015 Lausanne, Switzerland; 4 Department of Biochemistry, University Complutense Madrid, 28040 Madrid, Spain; 5 Bioinformatics Competence Centre, University of Lausanne, 1015 Lausanne, Switzerland; 6 Bioinformatics Competence Centre, École Polytechnique Fédérale de Lausanne, 1015 Lausanne, Switzerland; 7 Histology Core Facility, École Polytechnique Fédérale de Lausanne, 1015 Lausanne, Switzerland

**Keywords:** lung-on-chip, *Mycobacterium tuberculosis*, cords, biofilms, agent-based model, antibiotic therapy, mechanobiology, mycomembrane, serial block scanning face electron microscopy

## Abstract

*Mycobacterium tuberculosis* (Mtb) cultured axenically without detergent forms biofilm-like cords, a clinical identifier of virulence. In lung-on-chip (LoC) and mouse models, cords in alveolar cells contribute to suppression of innate immune signaling via nuclear compression. Thereafter, extracellular cords cause contact-dependent phagocyte death but grow *intercellularly* between epithelial cells. The absence of these mechanopathological mechanisms explains the greater proportion of alveolar lesions with increased immune infiltration and dissemination defects in cording-deficient Mtb infections. Compression of Mtb lipid monolayers induces a phase transition that enables mechanical energy storage. Agent-based simulations demonstrate that the increased energy storage capacity is sufficient for the formation of cords that maintain structural integrity despite mechanical perturbation. Bacteria in cords remain translationally active despite antibiotic exposure and regrow rapidly upon cessation of treatment. This study provides a conceptual framework for the biophysics and function in tuberculosis infection and therapy of cord architectures independent of mechanisms ascribed to single bacteria.

## Highlights

• Individual *M. tuberculosis* bacteria grow into high-aspect-ratio cords in alveolar cells
• Compression of host cell nuclei by mechanically resilient cords impairs immune responses
• Cords penetrate between cells, enabling bacterial dissemination to new tissue niches
• Tight packing of bacteria within cords confers protection from antibiotic clearance

## Graphical abstract

**Figure:**
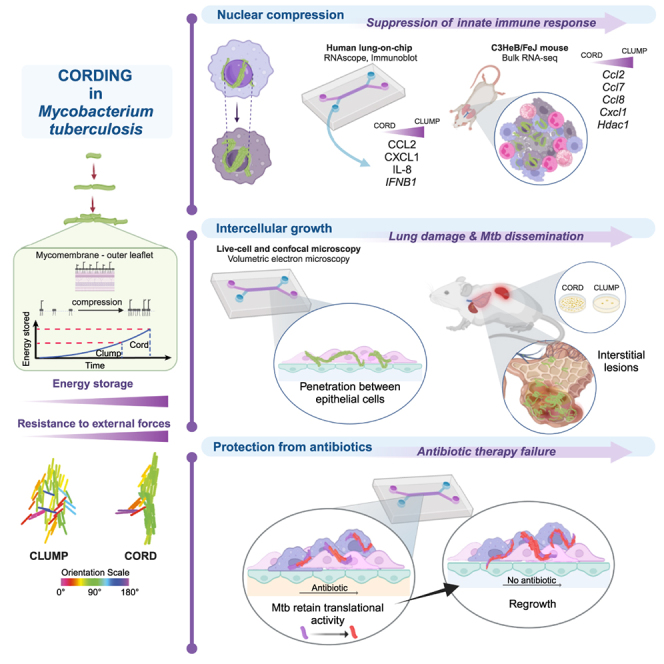

## Teaser

Energy storage in the cell membrane, made possible by the compression of cell surface lipids, enables individual *Mycobacterium tuberculosis* to grow into high-aspect-ratio mechanically resilient supracellular architectures known as cords. Tightly packed bacteria within cords are protected from antibiotic-mediated killing, dampen inflammatory responses, and enable bacterial dissemination by exerting forces on cellular organelles and tissues.

## Introduction

**Figure 1:**
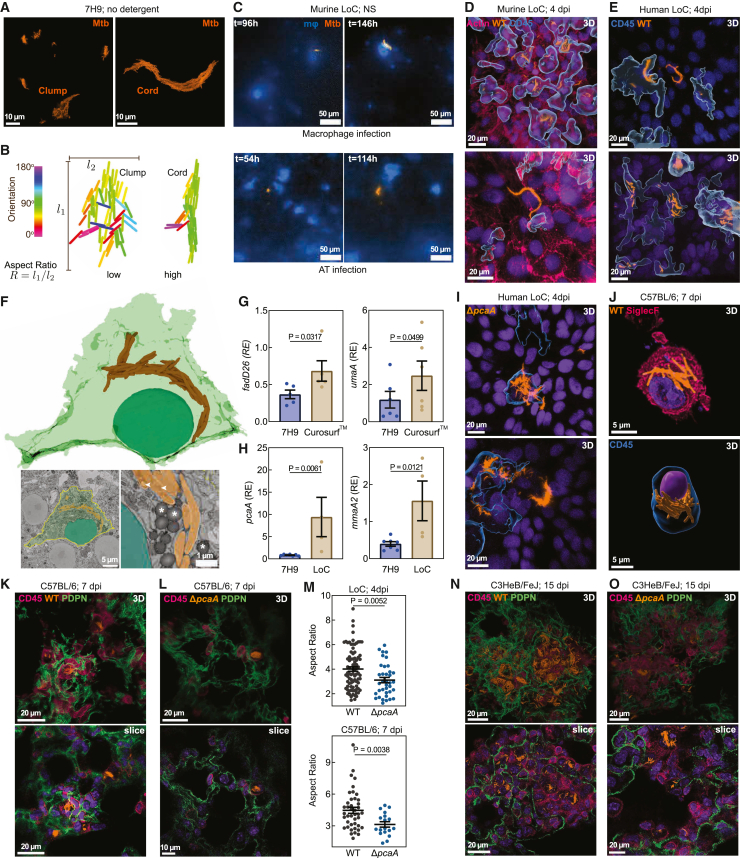
Air-liquid-interface-induced mycolic acid remodeling causes intracellular Mtb cord growth in early infection (A) Snapshots of Mtb architectures in detergent-free axenic culture. (B) Classification of Mtb architectures. (C) Time-lapse snapshots of WT cord growth in macrophages (top) and ATs (below) in surfactant-competent murine LoCs. NS, normal surfactant levels. (D and E) Confocal images of intracellular WT cords in macrophages (D, top, and E) and an AT (D, bottom, and E) in murine (D) and human (E) LoCs. (F) Representative volumetric EM images and 3D reconstruction of a WT cord in a murine AT at 4 dpi. Cell boundary (yellow line). Zooms: lipid inclusions (arrowheads), lipid droplets (asterisk) in Mtb (amber). (G and H) Expression levels of indicated genes in Mtb cultured axenically with or without exposure to Curosurf (G, n = 5) and in human LoCs at 3 dpi (H, n = 4). RE, relative expression. (I) Confocal images of Δ*pcaA* clumps in macrophages (top) and an AT (bottom) in human LoCs. (J) Representative images of WT cords in alveolar macrophages (AMs) explanted from C57BL/6 mice at 7 dpi. SiglecF (pink), CD45 (blue), and nucleus (purple). (K, L, N, and O) Representative slices and 3D views of intracellular WT cords (K and N) and Δ*pcaA* clumps (L and O) in AMs of C57BL/6 mice at 7 dpi (K and L) and C3HeB/FeJ mice at 15 dpi (N and O). CD45 (pink) and PDPN (green). (M) Aspect ratios for WT cords and Δ*pcaA* clumps (n > 42 from LoCs, n > 18 from mice). See also Figure S1 and Video S1.

*Mycobacterium tuberculosis* (Mtb), the causative agent of tuberculosis (TB), is typically associated with an intracellular lifestyle. Yet, the identification, several decades ago,^1^ of the growth of serpentine, high aspect ratio “cords” in liquid culture (Figure 1A) in the absence of detergents or as pellicles at the air-liquid interface (ALI)^2^ as characteristic of virulent strains^3^ suggests a role for these biofilm-like architectures in pathogenesis. Although cord formation is a conserved feature across pathogenic *Mycobacterium* spp.,^4^ reports on the direct manifestation of cording in Mtb pathogenesis have been sparse, unlike *Mycobacterium abscessus*^5^^,^^6^ or *Mycobacterium marinum*^7^ infections in the zebrafish model where extracellular cord growth is frequently reported. Mtb cords were shown to form in epithelial^4^^,^^8^ and fibroblast cell lines^9^ *in vitro* and were recently identified in lymphatic endothelial cells in patients with TB.^8^ However, the spatiotemporal sequence of cord formation and their biogeography within lesions remain poorly understood.^10^^,^^11^

Lipids in the mycomembrane—the outer membrane of the mycobacterial cell wall—are both direct effectors of pathogenesis and essential for cord formation.^12^ These include the complex mix of trehalose 6,6′-dimycolates (TDMs),^13^ also known as cord factor, with well-characterized conformation-dependent immunostimulatory effects.^14^^,^^15^ Compositional or conformational changes in mycomembrane lipids can also disrupt cord formation, but why Mtb cell wall lipids generate biofilm-like architectures with long-range bacterial alignment and high aspect ratios^16^ (Figure 1B), and the relevance of these architectures themselves for pathogenesis, remain relatively unknown.

Alveolar macrophages (AMs) provide a permissive environment for Mtb growth via fatty acid oxidation, and mycolic acid synthesis is highly upregulated during early infection.^17^^,^^18^ This led us to posit that cording may occur in the relatively understudied early stages of infection, which is well suited to lung-on-chip (LoC) models of early infection in the naive alveolar space.^19^

## Results

### Mtb cording is prominent in early infection

**Figure S1:**
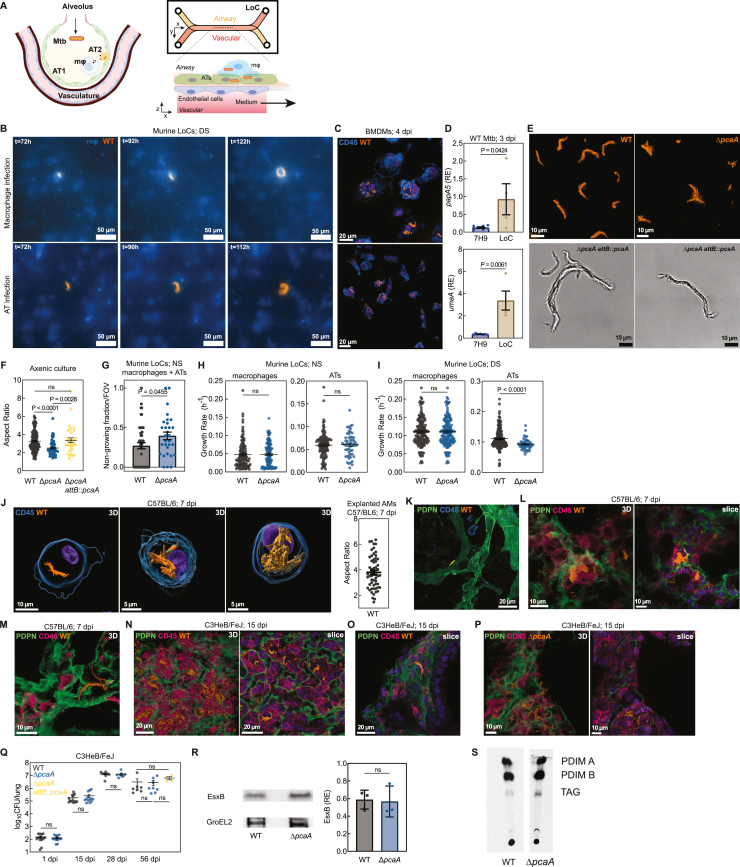
Mtb cording in the LoC and mouse models, related to Figure 1 (A) Schematic of LoC models. (B) Time-lapse snapshots of WT cords in macrophages (top) and ATs (below) in surfactant-deficient (DS) murine LoCs. (C) Confocal images of WT-infected BMDMs at 4 dpi. CD45 (blue). (D) Expression levels of the indicated genes in Mtb cultured axenically and from human LoCs at 3 dpi (n = 4). RE, relative expression. (E and F) Snapshots (E) and aspect ratios (F) of WT, Δ*pcaA*, and Δ*pcaA attB::pcaA* Mtb microcolonies cultured in detergent-free 7H9 (n > 33). (G) Non-growing fraction per field of view (FOV) for WT- and Δ*pcaA*-infected murine LoCs (n > 30 FOVs from n = 2 LoCs). (H and I) WT and Δ*pcaA* microcolony growth rates in macrophages and ATs in murine LoCs reconstituted with NS ATs (H) and DS ATs (I) (n > 110 for WT and n > 49 for Δ*pcaA* from n = 2 LoCs). (J and K) 3D views of WT cords in explanted AMs (J) and maximum intensity projection of WT cords in explanted AT1 cells (K) from C57BL/6 lungs at 7 dpi. CD45 (blue) and PDPN (green). (L and M) 3D views (L and M) and representative slice (L) of WT cords intracellular in AMs (L) and an AT (M) in C57BL/6 lungs at 7 dpi. CD45 (pink) and PDPN (green). (N–P) 3D views and representative slices of intracellular WT cords (N and O) and Δ*pcaA* clumps (P) in AMs (N and O) and an AT (P) in C3HeB/FeJ lungs at 15 dpi. (Q) Lung CFUs from WT-, Δ*pcaA*-, and Δ*pcaA attB::pcaA* Mtb-infected C3HeB/FeJ mice at all time points in this study. ns, not significant (n > 4 mice). (R) EsxB production by WT and Δ*pcaA* relative to GroEL2. (S) PDIM production by WT and Δ*pcaA*.

We used live imaging in a recently reported murine LoC model^19^^,^^20^ (Figure S1A) to investigate the growth dynamics of single Mtb bacteria constitutively expressing tdTomato (hereinafter referred to as wild type [WT]) in first-contact alveolar epithelial cells (ATs) and macrophages (Figure 1C). Strikingly, by 5 days post-infection (dpi), cord formation was prominent in both cell types (Figures 1C and 1D; Video S1). This was independent of previously reported host-protective roles for pulmonary surfactant secreted by ATs,^19^ although surfactant deficiency led to more rapid Mtb growth (Figure S1B; Video S1). Mtb cords were also evident in both ATs and macrophages in infected human LoCs (Figure 1E). Volumetric electron microscopy (EM) in murine ATs (Figure 1F) highlighted the tight packing of aligned Mtb bacteria, consistent with the properties of cord architectures (Figure 1B), and confirmed that Mtb bacteria within intracellular cords have free access to the host cell cytosol, are often associated with host-derived lipid droplets, and themselves contain prominent lipid inclusions (Figure 1F).^21^

Video S1. Time-lapse imaging of WT cord growth in a macrophage (first and third quarters of video) and in an AT (second and fourth quarters of video) in murine LoCs reconstituted with ATs producing normal surfactant levels (first half of video) and deficient surfactant levels (second half of video), related to Figure 1 Scale bar: 50 μm.

Cord growth in macrophages under standard cell culture conditions is rare (Figure S1C). We examined if exposure to host components specific to the ALI, such as pulmonary surfactant, might trigger cord formation. Mtb upregulated genes required for cell wall remodeling and mycolic acid synthesis such as *fadD26* and *umaA* within 1 h of exposure to Curosurf (Figure 1G), consistent with prior reports.^22^ Mtb from human LoCs at 3 dpi also showed a broad trend of upregulation of Mtb cell wall synthesis genes (Figures 1H and S1D). These included the cyclopropane synthase *pcaA* required for synthesis of TDMs with *proximal* cyclopropane rings and upregulated in infected AMs at 2 dpi.^23^ The deletion of the *pcaA* gene leads to a well-characterized cording deficiency evident in altered colony morphology on agar plates^12^^,^^24^ and in architectures with reduced aspect ratio in detergent-free axenic culture (Figure S1E). This phenotype was retained in murine and human LoCs, where Mtb Δ*pcaA* constitutively expressing tdTomato (hereinafter referred to as Δ*pcaA*) formed disordered clumps (Figure 1I) with significantly reduced aspect ratio (Figures 1M, top, and S1F). In axenic culture, complementation of the *pcaA* gene effectively restores the cording phenotype (Figures S1E and S1F).

Live imaging in surfactant-competent murine LoCs showed a greater proportion of non-growing^19^ Δ*pcaA* (Figure S1G), reinforcing the importance of cyclopropane modification of TDM in surviving first contact with the host. However, microcolonies that grew out of individual WT and Δ*pcaA* bacteria that survived first contact had similar growth kinetics (Figures S1H and S1I).

This strong signal for early cord formation in LoCs prompted us to identify if such architectures are also present in early infection *in vivo*. First, we exhaustively characterized explanted pulmonary cells from C57BL/6 mice at 7 dpi via microscopy. The majority of infected cells were CD45^+^ SiglecF^+^ AMs,^19^^,^^25^ and many of these cells had notable cord formation (Figures 1J and S1J). Instances of cords in podoplanin (PDPN^+^) type 1 ATs were also documented (Figure S1K). We then performed high-resolution confocal imaging of 150-μm-thick fixed frozen lung slices to identify and characterize these architectures directly *in situ*. At 7 dpi, we identified cords in CD45^+^ cells located in alveoli that morphologically resembled AMs (Figures 1K and S1L) and in PDPN^+^ type I ATs lining the alveoli (Figure S1M), consistent with the data from explanted cells. Importantly, infection with Δ*pcaA* also led to formation of disordered clumps and not cords in first-contact host cells in C57BL/6 mice (Figures 1L and 1M, bottom). These phenotypes and morphological observations were also evident in C3HeB/FeJ mice, which are more permissive to Mtb growth than C57BL/6 mice and develop lesions that more closely resemble the immunopathology of human disease^26^ (Figures 1N, 1O, and S1N–S1P). In C3HeB/FeJ mice, there was no difference in pulmonary bacterial burden between the two strains at all time points (Figure S1Q), in agreement with comparable levels of effectors of Mtb pathogenesis such as ESX-1^27^^,^^28^ and phthiocerol dimycocerosates (PDIMs)^29^^,^^30^ in both strains (Figures S1R and S1S). Together, these results establish cord formation as a prominent feature of early pathogenesis in the alveolar space *in vivo*, which is effectively captured in more tractable LoC models.

### Intracellular cording suppresses immune signaling

**Figure S2:**
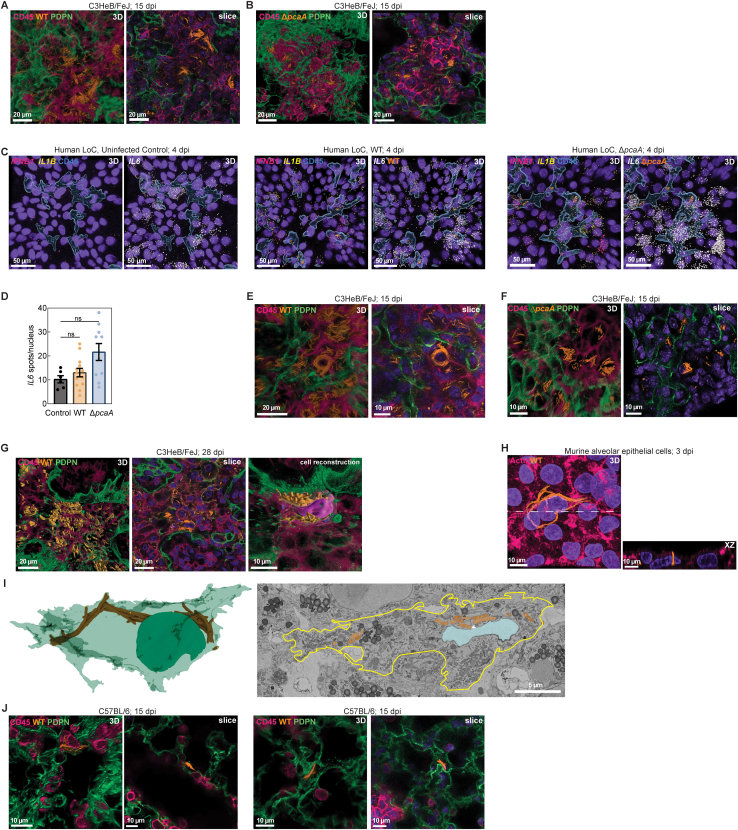
Nuclear compression and hypoinflammatory responses due to Mtb cording, related to Figure 2 (A and B) 3D views and representative slices of foci of infection in (A) WT- and (B) Δ*pcaA*-infected C3HeB/FeJ lungs at 15 dpi. CD45 (pink) and PDPN (green). (C) Representative 3D views from the epithelial face of WT- and Δ*pcaA*-infected human LoCs at 4 dpi. *IFNB1* (pink), *IL1B* (yellow), *IL6* (gray), and CD45 (blue surfaces). (D) RNAscope characterization of *IL-6* levels from the epithelial face of WT- and Δ*pcaA*-infected human LoCs (n > 6 FOVs from n = 2 LoCs) at 4 dpi. (E and F) 3D views and representative slices of heavily infected AMs in (E) WT- and (F) Δ*pcaA*-infected C3HeB/FeJ lungs at 15 dpi. CD45 (pink) and PDPN (green). (G) 3D views, representative slice, and reconstruction of a heavily infected AT in WT-infected C3HeB/FeJ lungs at 28 dpi. (H) 3D view and XZ orthosection of an intracellular WT cord in murine AT cell monolayers at 3 dpi. (I) Representative volumetric EM images and 3D reconstruction of a WT cord in a murine AT at 4 dpi. Cell boundary (yellow line), white arrowhead: direct contact between nucleus and cord. (J) 3D views and representative slices of infections of AT cells in WT-infected C57BL/6 lungs at 15 dpi.

**Figure 2:**
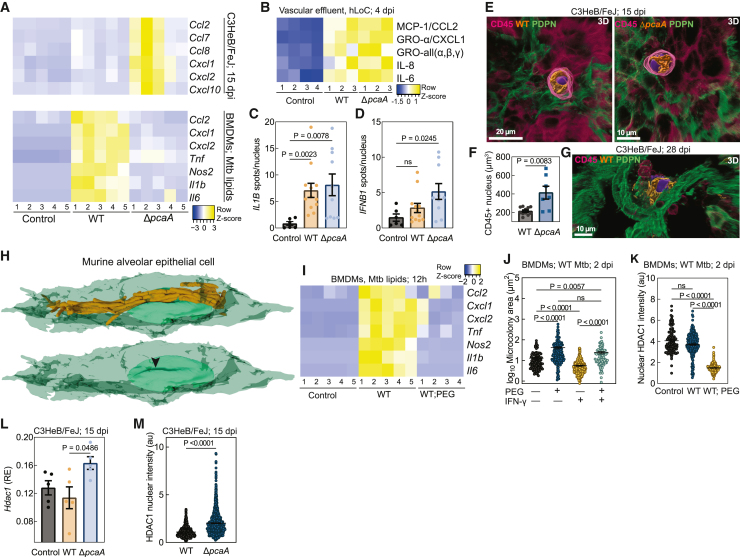
Sustained compression of host cell nuclei by intracellular Mtb cords leads to HDAC1-mediated immunosuppression (A) Transcriptional response of WT- and Δ*pcaA*-infected C3HeB/FeJ lungs at 15 dpi (top), and BMDMs at 12 h post-exposure (hpe) to WT and Δ*pcaA* surface lipid monolayers (bottom). (B) Immunoblot assays on human LoC effluent collected up to 4 dpi. (C and D) RNAscope characterization of selected markers from the epithelial face of control, WT-, and Δ*pcaA*-infected human LoCs (n > 6 fields of view from n = 2 LoCs) at 4 dpi. (E) Representative 3D views of WT-infected (left) and Δ*pcaA*-infected (right) AMs within C3HeB/FeJ lungs at 15 dpi. CD45 (pink), PDPN (green), and nucleus (purple surface). (F) Nuclear volume of heavily infected AMs within WT- and Δ*pcaA*-infected C3HeB/FeJ lungs at 15 dpi (n > 8 from n = 2 mice). (G) Representative 3D view of a WT-infected AT cell within C3HeB/FeJ lungs at 28 dpi. (H) 3D reconstruction from volumetric EM of a WT cord indenting the cellular nucleus (arrowhead, bottom) in a murine AT cell at 4 dpi. (I) Transcriptional response of BMDMs at 12 hpe to WT surface lipids monolayers cultured with or without 10% PEG. (J) Mtb microcolony area in WT-infected BMDMs at 2 dpi with the combinatorial addition of 10% PEG and IFN-γ (n > 95). (K) Mean HDAC1 intensity in WT-infected BMDM nuclei cultured with or without 10% PEG (n > 179). (L) *Hdac1* expression in uninfected, WT-, and Δ*pcaA*-infected C3HeB/FeJ lungs at 15 dpi (n = 5 mice). (M) Mean nuclear HDAC1 intensity at foci of infection in WT- and Δ*pcaA*-infected C3HeB/FeJ lungs at 15 dpi (n > 895 from n = 3 mice). See also Figures S2 and S3.

We compared infections with WT and Δ*pcaA* in the LoC and the C3HeB/FeJ models to identify direct roles in pathogenesis for cord architectures. An examination of lung slices from C3HeB/FeJ mice at 15 dpi revealed a substantial influx of immune cells into the alveolar space in Δ*pcaA* infections. This was less evident in WT infection, where infected AMs were often located in the interstitial space (Figures S2A and S2B). Transcriptomic measurements from Δ*pcaA*-infected C3HeB/FeJ lungs showed upregulation of genes (*Ccl2* and *Cxcl10*) associated with an incipient TB signature in humans^31^^,^^32^ as well as stimulatory chemokines for monocyte and neutrophil infiltration (*Ccl7*, *Ccl8*, *Cxcl1*, and *Cxcl2*) (Figure 2A, upper), consistent with the observed pathology (Figures S2A and S2B). WT infections generated a consistently lower inflammatory response despite similar pulmonary bacterial burdens (Figure S1Q). This differential immune response was also evident in the human LoC model, where immunoblot analyses confirmed elevated levels of monocyte and neutrophil attractant chemokines (CCL2, CXCL1, GRO-α, GRO-β, GRO-γ, and interleukin-8 [IL-8]) in effluent collected over 1–4 dpi from Δ*pcaA*-infected vs. WT-infected LoCs (Figure 2B). RNAscope measurements (Figure S2C) confirmed that both WT and Δ*pcaA* infections elevated epithelial *IL1B* expression (Figure 2C), consistent with early IL-1R signaling in mouse models.^25^^,^^33^ *IFNB1* expression, a correlate with faster disease progression and greater tissue damage,^31^^,^^34^^,^^35^ was significantly elevated only in Δ*pcaA* infections (Figure 2D). Epithelial *IL6* expression was unchanged in infections with both strains, suggesting the elevated IL-6 levels in the effluent may arise from endothelial cell inflammation (Figures 2B, S2C, and S2D). Together, these results provide evidence of a conserved attenuated inflammatory response to WT but not Δ*pcaA* infections.

Previous studies have reported an opposite trend, i.e., an attenuated inflammatory response of murine bone marrow-derived macrophages (BMDMs) infected with Δ*pcaA* vs. WT, a direct consequence of the loss of proximal *cis*-cyclopropanation in Δ*pcaA* lipids.^24^ We confirmed these observations in BMDMs exposed to monolayers of WT and Δ*pcaA* surface lipids (Figure 2A, lower).^24^ This effectively ruled out differences in lipid composition between the two strains as the cause of the attenuated immune response to WT. Instead, we hypothesized a role for the cord architecture itself.

**Figure S3:**
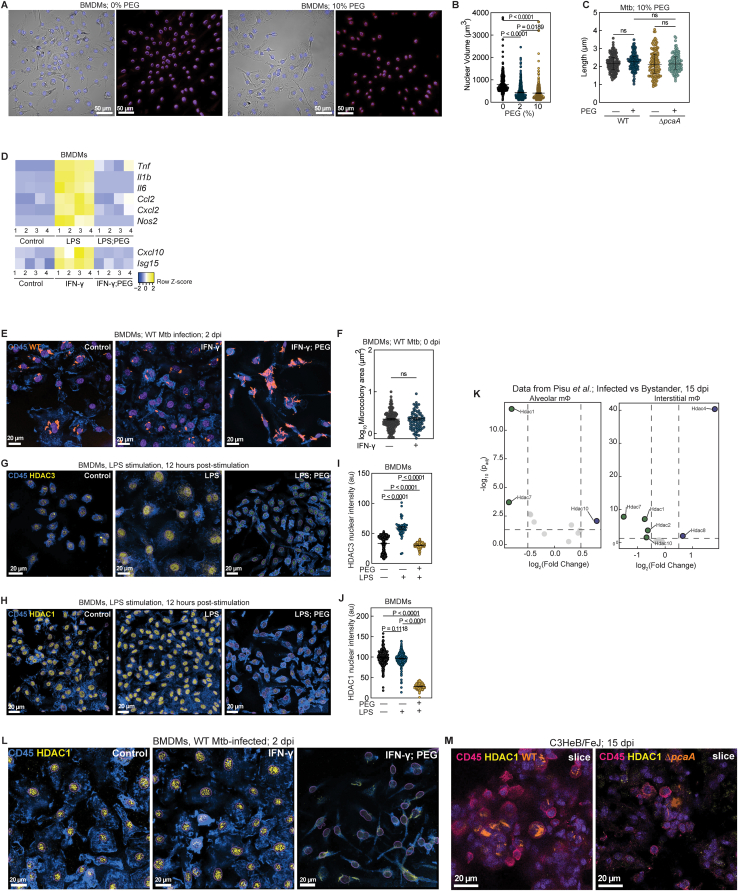
PEG-induced compression attenuates inflammatory responses and HDAC1 expression in early TB, related to Figure 2 (A and B) Representative maximum intensity projections and 3D views of BMDM nuclei (A) and nuclear volume (B) cultured with or without 10% PEG (n > 550). (C) Cell length of WT and Δ*pcaA* (n = 125) cultured axenically with or without 10% PEG. (D) Transcriptional response of BMDMs at 12 hpe to LPS and IFN-γ cultured with or without 10% PEG. (E) Representative images of WT-infected BMDMs at 2 dpi with the combinatorial addition of 10% PEG and IFN-γ. (F) Microcolony area in WT-infected BMDMs with or without IFN-γ pre-exposure at 0 dpi (n > 80). (G–J) Representative 3D views (G and H) and expression of HDAC3 (I) and HDAC1 (J) in BMDMs 12 hpe to LPS cultured with or without 10% PEG. HDAC3 (G, yellow), HDAC1 (H, yellow), and CD45 (blue) (n > 41). (K) *Hdac1-9* expression in infected AMs and interstitial macrophages relative to uninfected bystanders at 15 dpi. Data from Pisu et al.^18^ (L) Representative images of WT-infected BMDMs pre-stimulated with IFN-γ cultured with or without 10% PEG. CD45 (blue) and HDAC1 (yellow). (M) Representative slices from foci of infection in WT- and Δ*pcaA*-infected C3HeB/FeJ lungs at 15 dpi. CD45 (pink) and HDAC1 (yellow).

*In situ* confocal imaging of heavily infected CD45^+^ cells in C3HeB/FeJ lungs at 15 dpi revealed that whereas both Δ*pcaA* clumps and WT cords occupied a significant fraction of the cytoplasmic volume, only WT cords enveloped the cellular nucleus (Figures 2E, S2E, and S2F). This correlated with reduced nuclear volume in WT-infected CD45^+^ cells (Figure 2F). Similar interactions were also observed in infected ATs (Figure 2G). In some instances, the cell nucleus was shaped and constrained by the cord architecture (Figures 2G and S2G). This was confirmed via volumetric EM imaging of ATs infected *in vitro*, where direct contact between bacteria in an intracellular WT cord and the nuclear membrane, and the resulting clear indentation of the nucleus, was evident (Figures 2H, S2H, and S2I). Mechanical constriction of immune cell nuclei (e.g., via cellular constriction) has been shown to attenuate responses to pro-inflammatory stimuli, mediated by altered chromatin accessibility and histone deacetylase (HDAC) activity.^36^^,^^37^ We considered if similar mechanisms may operate in heavily infected cells with cord architectures. Notably, sites of AT infection *in vivo* often had reduced immune infiltration (Figures 2G and S2J), consistent with this hypothesis. To obtain mechanistic insight, we induced nuclear compression *in vitro* in BMDMs via polyethylene glycol (PEG)-induced cytoplasmic crowding^38^ (Figures S3A and S3B), which had no effect on Mtb cell length (Figure S3C). This PEG-compression model replicated the attenuation of pro-inflammatory responses to lipopolysaccharide (LPS) and interferon-γ (IFN-γ) stimulation^36^ (Figure S3D). PEG compression also attenuated BMDM responses to WT surface lipids (Figure 2I) and reduced the ability of both resting and IFN-γ pre-activated BMDMs to control Mtb growth (Figures 2J, S3E, and S3F).

We sought to identify epigenetic regulators of these transcriptional responses, focusing on class I HDACs that have effector functions in the nucleus. Resting BMDMs exposed to LPS in the presence of PEG showed reduced nuclear HDAC3 levels (Figures S3G and S3I), consistent with previous reports.^36^ Additionally, we observed reduced nuclear HDAC1 levels (Figures S3H and S3J). Recent dual RNA-seq data^18^ showed reduced *Hdac1* expression in Mtb-infected alveolar and interstitial macrophages (Figure S3K). We confirmed a signature of reduced HDAC1 nuclear localization (Figure 2K) and *Hdac1* expression (Figure S3L) in WT-infected BMDMs exposed to PEG. Interestingly, *Hdac1* expression was elevated in Δ*pcaA-*infected C3HeB/FeJ lungs at 15 dpi relative to the lungs of WT-infected mice and uninfected controls (Figure 2L), which also correlated with an increased nuclear HDAC1 intensity in tissue sections (Figures 2M and S3M). Thus, despite the greater inflammatory nature of WT surface lipids, intracellular mechanical interactions between Mtb cords and host cell organelles contribute to the suppression of early inflammatory signaling in TB.

### Cording deficiency reduces bacterial dissemination from alveolar space

**Figure 3:**
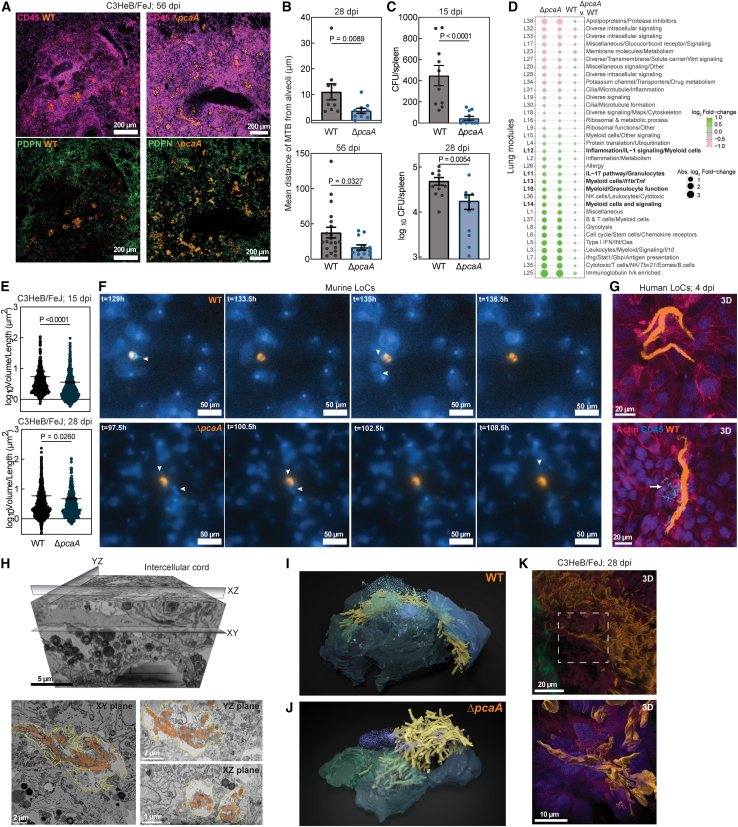
Intercellular Mtb cord growth facilitates Mtb dissemination with reduced tissue inflammation (A) Representative tile scans of WT-infected (left) and Δ*pcaA*-infected (right) C3HeB/FeJ lungs at 56 dpi. CD45 (pink) and PDPN (green). (B) Mean distance of WT and Δ*pcaA* lesions from alveoli in C3HeB/FeJ lungs (n > 10 scans from n = 3 mice) at 28 (top) and 56 dpi (bottom). (C) CFUs from WT- and Δ*pcaA*-infected C3HeB/FeJ spleens at 15 and 28 dpi (n > 10 mice). (D) Modular transcriptional analysis of WT- and Δ*pcaA*-infected C3HeB/FeJ lungs (n = 8 mice) at 56 dpi. Green (over-abundant) and pink (under-abundant) modules, compared with controls (n = 6 mice); color intensity and dot size represent degree of perturbation; false discovery rate (FDR) < 0.05 considered significant. (E) Volume to binding box length ratio for WT and Δ*pcaA* aggregates in C3HeB/FeJ lungs (n > 671 from n = 4 mice) at 15 and 28 dpi. (F) Time-lapse snapshots of WT (top) and Δ*pcaA*-infected murine LoCs. Arrowheads: macrophages interacting with Mtb aggregates, GFP macrophages (blue). (G) Confocal images from a human LoC showing WT cords intercellular between AT cells. Arrow: dying macrophage (bottom). Actin (pink) and CD45 (blue). (H) 3D volumetric EM of an intercellular WT cord (amber) between the plasma membranes of adjacent cells (yellow) in XY, YZ, and XZ slices. (I and J) 3D reconstruction of a WT intercellular cord (I) in between live AT cells and a Δ*pcaA* clump on top of live AT cells (J). Sparkles: debris of the original host cells. (K) 3D views of an intercellular WT cord in a C3HeB/FeJ lung at 28 dpi. CD45 (pink), PDPN (green), and nucleus (purple). See also Figure S4 and Video S2.

**Figure S4:**
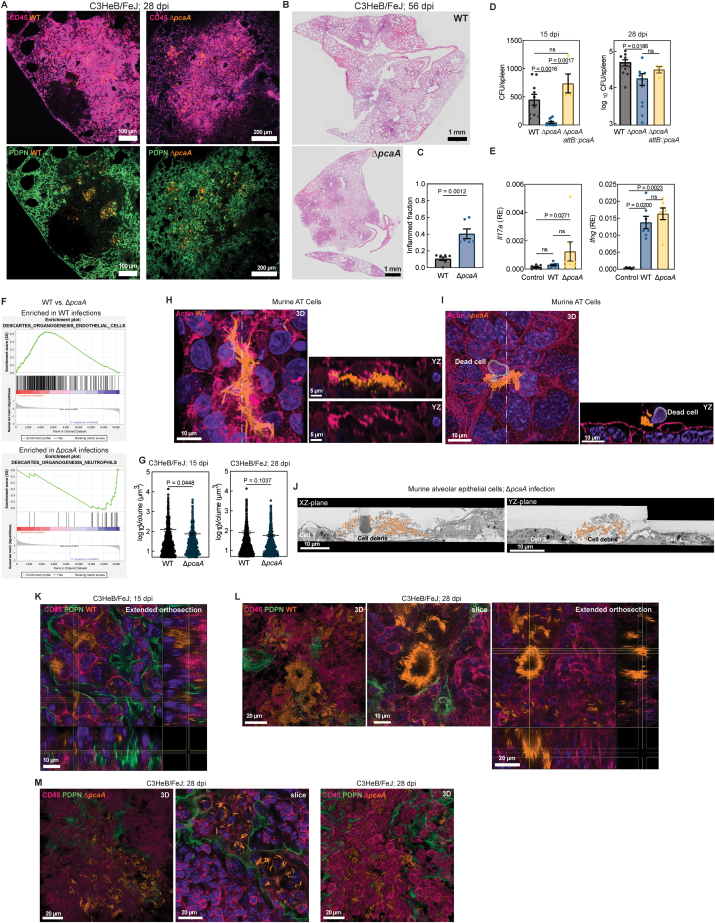
Immunopathological differences between WT- and Δ*pcaA*-infected lesions, related to Figure 3 (A) Representative tile scans of WT- and Δ*pcaA*-infected C3HeB/FeJ lungs at 28 dpi. CD45 (pink) and PDPN (green). (B and C) Representative images and fractional area of inflammation from hematoxylin and eosin (H&E)-stained WT- and Δ*pcaA*-infected C3HeB/FeJ lungs at 56 dpi. (n > 6 FOV from n = 3 mice.) (D) CFUs from C3HeB/FeJ spleens infected with WT, Δ*pcaA*, and Δ*pcaA attB::pcaA* at 15 and 28 dpi (n > 3 mice). (E) Expression levels of indicated genes in WT- and Δ*pcaA-*infected C3HeB/FeJ lungs at 56 dpi (n > 6 mice). (F) GSEA running enrichment score plot between WT- and Δ*pcaA-*infected C3HeB/FeJ mice at 56 dpi (n = 8 mice). (G) Mtb aggregates’ volume in WT- and Δ*pcaA-*infected C3HeB/FeJ lungs at 15 and 28 dpi (n > 671 from n = 4 mice). (H) 3D views and YZ orthosections of an intercellular WT cord in a murine AT monolayer, with (top) or without the cord shown (bottom). Actin (pink) and nucleus (purple). (I) 3D views and YZ orthosections of a Δ*pcaA* clump above an AT monolayer. Nuclear debris of the original host cell (gray surface). (J) XZ and YZ slices from 3D volumetric EM image of the Δ*pcaA* clump (amber) in Figure 3J. (K) Extended 2D views of an intercellular cord in WT-infected C3HeB/FeJ lungs at 15 dpi. CD45 (pink) and PDPN (green). (L) 3D and extended 2D views and a representative slice of an intercellular cord at the center of a WT lesion in C3HeB/FeJ lungs at 28 dpi. (M) 3D views and a representative slice of Δ*pcaA* lesions in C3HeB/FeJ lungs at 28 dpi.

Next, we sought to examine how the early formation of intracellular Mtb cords influences the subsequent host-pathogen interactions that lead to the onset of adaptive immunity (28 dpi) and the start of chronic disease (56 dpi) in the C3HeB/FeJ model. In a previous comparative study following intravenous Mtb administration in C57BL/6 mice,^12^ Δ*pcaA* showed a long-term persistence defect with significantly lower burden at 135 dpi and attenuation in a time-to-death analysis at 219 dpi, relative to WT. In C3HeB/FeJ mice, there were clear pathological differences in the architecture of inflammatory lesions despite no significant difference in bacterial lung burdens at 28 and 56 dpi (Figure S1Q). Bacteria in WT lesions were at the core of well-formed granulomatous architectures predominantly located in interstitial spaces away from otherwise uninflamed alveolar areas (Figures 3A, 3B, and S4A). In contrast, a greater fraction of infection foci in Δ*pcaA*-infected mice were localized in the alveolar space, often leading to alveolitis and pneumonia that spread between adjacent alveoli (Figures 3A, 3B, and S4A). A histological analysis confirmed a notable increase in immune infiltration in Δ*pcaA* infections (Figures S4B and S4C). Notably, these differences in lesional architectures correlated with a reduced dissemination to the spleen of Δ*pcaA* vs. WT at 15 and 28 dpi (Figure 3C), which was not evident in infections with Δ*pcaA attB::pcaA* Mtb at both time points (Figure S4D).

Bulk RNA-seq analyses of infected lungs confirmed that infection with both strains generated a strong inflammatory response evident in histological observations at 56 dpi (Figure 3D). We used the recently introduced mouse lung module framework^39^ to characterize the differences between the two strains (Figure 3D). The response to Δ*pcaA* infection was skewed toward a stronger Th17 response (module L11 in Figures 3D and S4E) and an increased expression of genes related to myeloid and granulocyte signaling (modules L10, L13, and L14). Both strains generated robust type II IFN responses (module L7 in Figures 3D and S4E). A comparative gene set enrichment analysis (GSEA) for cell-type signature gene sets identified an enrichment for neutrophils in Δ*pcaA* infections, consistent with lung module framework characterization and an enrichment for endothelial cells in WT infections, consistent with the pro-angiogenic role of cyclopropanated mycolic acids^40^ (Figure S4F).

Next, we sought to determine the role of cord architectures themselves in driving these physiological changes. The total volumes of individual bacterial microcolonies in WT infections were significantly elevated over Δ*pcaA* at 15 dpi and remained elevated at 28 dpi (Figure S4G). The ratio of volume to the greatest dimension of the bounding box that fit each microcolony was significantly higher for WT infections at all time points, consistent with greater bacterial density expected in Mtb cords (Figure 3E).

The sequence of host-pathogen interactions is difficult to ascertain through examination of lung slices at fixed time points. We used the LoC model to examine interactions of macrophages and AT cells with cords released into the extracellular space subsequent to the death of the “first contact” host cell. On-chip, both WT cords and Δ*pcaA* clumps emerging from dead “first contact” cells were strikingly toxic to macrophages (Figures 3F and 3G; Video S2). Macrophage cell death resulted rapidly from attempted phagocytosis^41^ in many instances occurring directly after contact without bacterial uptake. This delinks aggregate-induced cell death through ESX-1- and PDIM-mediated damage to the plasma membrane^42^^,^^43^ from the architecture of these aggregates. Interactions with large Mtb aggregates have striking cell-type-specific consequences—neither architecture caused rapid cell death when in contact with adjacent AT cells.

Video S2. Time-lapse imaging of contact-dependent cell death of macrophages interacting with WT cords (first half of video) and Δ*pcaA* clumps (second half of video) in a murine LoC reconstituted with ATs producing normal surfactant levels, related to Figure 3 Scale bar: 50 μm.

Curiously, time-lapse imaging suggested that in the relative absence of phagocytic attempts by macrophages, Mtb cords appeared to span several epithelial cells but co-localized with actin staining, which suggested an intracellular localization (Figures 3G and S4H). To resolve this, we performed correlated optical microscopy and volumetric EM on cord architectures that spanned several epithelial cells in Mtb-infected AT cell monolayers. Unexpectedly, the Mtb cord had grown out of the necrotic cell debris of the original host cell^44^ in between the tight junctions of adjacent epithelial cells (Figures 3H and 3I), but monolayer integrity was retained by junctions between adjacent cells that were intact above and below the cord (YZ and XZ planes in Figure 3H). This radical mode of intercellular growth explained the co-localization observed with the actin staining across multiple epithelial cells, as the axial resolution of light microscopy is insufficient to identify the presence of the cord between cellular junctions. In contrast, Δ*pcaA* bacteria (Figures 3J, S4I, and S4J) were unable to penetrate between AT cells but rather grew as a clump within the necrotic debris of the original host cell. Cord architectures thus enable individual growing bacteria to collectively exert a force on their surroundings, in a manner that a clump of bacteria cannot.

Immune cell influx makes the identification of dynamic cord architectures challenging *in vivo*. However, instances of intercellular cords were evident in WT lesions (Figures 3K and S4K), often located at the core of developing areas of necrosis (Figure S4L). In contrast, Δ*pcaA* lesions predominantly contained intracellular bacteria (Figure S4M). Cord-like architectures are evident at the onset of the adaptive immune response, consistent with observations of cords in lymphatic endothelial cells.^8^ Intercellular and extracellular cord growth at early to intermediate stages of infection shapes the biogeography of lesion formation, causes tissue necrosis, and enables Mtb dissemination at later stages, which are necessary for cavitary lesion formation and Mtb transmission. These pathological changes are orchestrated by the mechanical effect of rigid cord architectures acting in synergy with other effectors of Mtb pathogenicity.

### Lipid compressibility determines cord architecture

**Figure 4:**
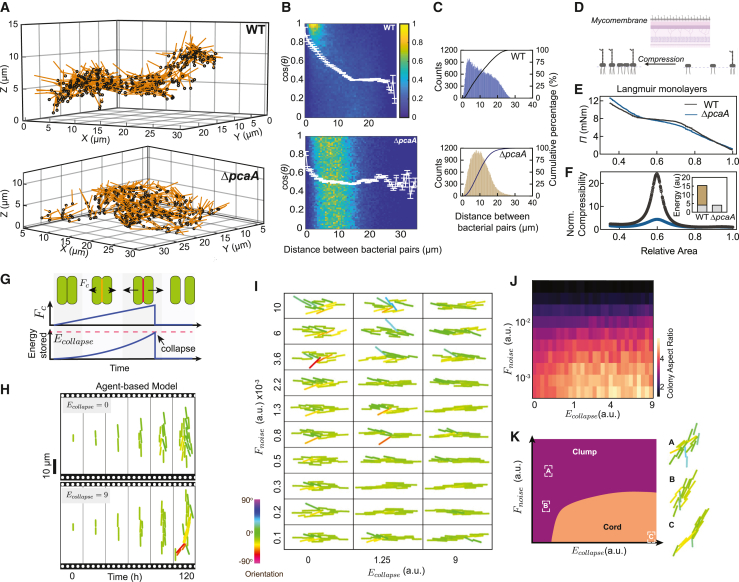
Surface lipid compressibility explains tight-packing and long-range order in Mtb cords (A) 3D vector plot of an intercellular WT cord and an extracellular Δ*pcaA* clump from Figures 3I and 3J. (B) 2D-binned histogram of the alignment (cosθ) vs. distance between randomly chosen bacterial pairs. White line: <cosθ>. (C) Histogram and cumulative probability curves of distance from (B). (D–F) Schematic (D) and plots of (E) surface pressure Π and (F) normalized compressibility vs. relative area for Langmuir monolayers of WT and Δ*pcaA* surface lipids (n = 3 technical replicates). (F, inset) Energy stored: reversible work (gray) and latent heat (green). (G) Schematic of interaction forces and $E_{collapse}$ in the agent-based model. (H) Simulated microcolony growth for low and high $E_{collapse}$. (I) Simulated microcolonies with a sweep of $E_{collapse}$ and $F_{noise}$ applied during growth. (J) Phase diagram (n = 20 simulations) of the median colony aspect ratio as a function of $E_{collapse}$ and $F_{noise}$. (K) Classification of microcolony architectures into cords and clumps. See also Figure S5 and Video S3.

**Figure S5:**
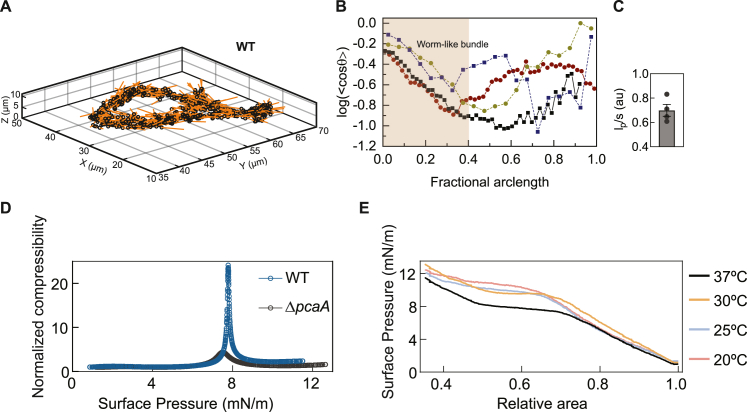
Worm-like bundle model for WT cords, related to Figure 4 (A) 3D vector plot of the intercellular WT cord in Figure 3H. (B) Log (<cosθ>) vs. fractional arclength along an XY projection. Dashed lines: intracellular cords in Figures 1D and S2I; solid lines: intercellular cords in Figure 3I and in (A). (C) Persistence length to arclength ratio lps, obtained from (B). (D) Normalized compressibility vs. surface pressure for Langmuir monolayers of WT and Δ*pcaA* surface lipids. (E) Π−A isotherms for WT lipids at different temperatures (n = 3 technical replicates in D and E).

The direct intracellular and extracellular application of forces by cords prompted us to examine the mechanisms that confer mechanical resilience to cords. We annotated the end-to-end coordinates of each bacterium in the high-resolution volumetric EM dataset in Figures 3H–3J as vectors and generated a spatial map of each bacterium in two WT cords and one Δ*pcaA* clump (Figures 4A and S5A). A 2D histogram of the normalized scalar product cosθ of all pairwise combinations of individual bacteria vs. interbacterial distance demonstrates the alignment of the bacteria within WT cords over several bacterial lengths, which is absent in the Δ*pcaA* clump (Figure 4B). This is also reflected in the tighter packing of bacteria within WT cords (Figure 4C). Instantaneously, WT cords can be described as worm-like bundles in the shear-dominated regime,^45^ with persistence length *l*_*p*_ that scales with cord length *s* (Figures S5B and S5C). This indicates that these architectures are held together by strong interbacterial forces.

The outer leaflet of the mycomembrane is considered to be mechanically independent from underlying arabinogalactan cell wall components.^46^^,^^47^ We hypothesized that hydrophobic interactions between the lipids in the outer leaflet of the mycomembrane of bacteria lying side by side in a cord might generate sufficient adhesive forces to maintain bacterial alignment in the cords. The loss of proximal *cis*-cyclopropanation in Δ*pcaA* reduces the proportion of TDMs containing compressible alpha and methoxy mycolic acids and increases the proportion of TDMs containing relatively brittle keto mycolic acids,^48^ supporting this hypothesis. Furthermore, there were no detectable differences between the two strains in other components of the mycomembrane including PDIMs and ESX-1 proteins (Figures S1R and S1S), nor evidence from the volumetric EM to suggest the presence of a capsule layer in between apposed bacteria.

The biophysical properties of alpha, keto, and methoxy mycolic acids have only been studied in isolation experimentally.^48^^,^^49^ We examined Langmuir monolayers^50^ of the total surface lipids extracted from pellicle biofilms^2^ of the WT and Δ*pcaA* strains (Figure 4D). At 37°C, the isotherm of surface pressure Π vs. relative surface area for WT lipids shows a first-order transition from a liquid-expanded to a liquid-condensed phase, which is not evident in the Δ*pcaA* isotherm (Figure 4E). Monolayer collapse occurred at Π∼12mN/m for lipids from both strains. WT lipids are significantly more compressible owing to the conformational change in lipids during the phase transition (Figures 4F and S5D) and store 4-fold more energy in the transition from gaseous state to monolayer collapse (Figures 4F and S5E; STAR Methods).

We developed an agent-based simulation for Mtb growth^51^^,^^52^ to build on the intuition gained from the Langmuir monolayer experiments and to visualize how energy storage in the membrane might drive cord-like growth (STAR Methods). The model has one free parameter—the collapse energy $E_{collapse}$ defined as the maximum potential energy stored between two bacteria in physical contact but pulled away from each other, prior to separation of the two cells (Figure 4G). The model excludes quorum sensing or other signaling interactions between bacteria. Thermal noise and forces applied by the cellular environment on the bacteria are represented by Gaussian noise $F_{noise}$ applied to each bacterium at each time point. We used colony aspect ratio (Figure 1) to compare the morphology of simulated microcolonies with experimental datasets for two values of $E_{collapse}$ with low $F_{noise}$. Remarkably, the simulated growth of bacteria with low $E_{collapse}$forms microcolonies that are disorganised, low aspect ratio clumps, whereas high $E_{collapse}$generated high aspect ratio cords (Figure 4H; Video S3). These results validate our intuition that an increased ability to store energy during cell-to-cell interaction (i.e., increased $E_{collapse}$) is sufficient by itself to lead to cord formation.

Video S3. Simulated growth of an Mtb microcolony starting from a single bacterium with *E*_collapse_ = 0 (first half of video) and with *E*_collapse_ = 0 a.u. (second half of video), related to Figure 4

Next, we used the agent-based model to vary both varying $E_{collapse}$ and the $F_{noise}$ applied to the bacteria during growth (Figure 4I) to identify conditions for growth of bacterial microcolonies into cords or clumps. The resulting phase diagram of aspect ratio (Figure 4J) can be structured into two physiologically relevant regions: high ratios correspond to tightly packed architectures with long-range order (orange zone) resembling WT cords, and low and intermediate ratios correspond to loosely packed, locally ordered bacteria that form disordered bacterial aggregates (purple zone) resembling Δ*pcaA* clumps (Figure 4K).

### Mtb cords maintain structural integrity despite mechanical perturbations

**Figure 5:**
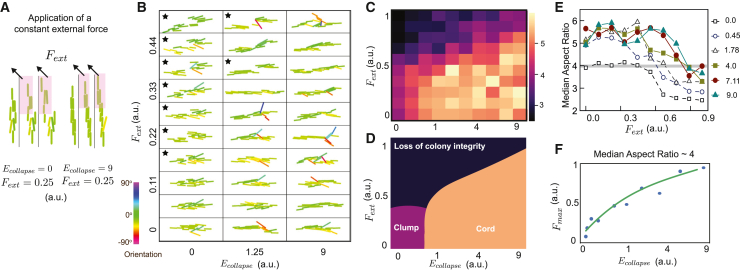
Cord architectures are resilient to mechanical perturbation and can exert forces (A) $F_{ext}$ applied to bacteria in the pink area of microcolonies with high and low $E_{collapse}$. (B) Simulated microcolonies with a sweep of $E_{collapse}$ and $F_{ext}$, applied during the last cell cycle. Black stars: microcolonies that separate into two. (C) Phase diagram of colony aspect ratio as a function of $E_{collapse}$ and $F_{ext}$. (D) Classification of microcolony architectures into cords and clumps. (E) Colony aspect ratio as a function of $F_{ext}$, for several values of $E_{collapse}$. (F) $F_{\max}$ as a function of $E_{collapse}$. Green line: spline fit. In (C)–(F), median values from n = 25 agent-based simulations. See also Video S4.

Next, we applied the agent-based model to explore how the microcolony architectures responded to mechanical perturbations such as a large, directed, external force. This mimics forces generated by interactions with cell junctions or during attempted phagocytosis (Figure 5A). After three generations of growth under low $F_{noise}$, a force $F_{ext}$ is applied for two interdivision periods to the bacteria at one end of the microcolony (Figure 5A). For $E_{collapse}=0\text{,}$ the clump quickly splits into two parts, losing its structural integrity and lowering its aspect ratio (Figure 5A, left; Video S4). On the contrary, for a high $E_{collapse}$, the cord remains intact (Figure 5A, right; Video S4).

Video S4. Simulated growth of an Mtb microcolony starting from a single bacterium, with *E*_collapse_ = 0 (first half of video) and *E*_collapse_ = 0 a.u. (second half of video), related to Figure 5 In each case, an external force is applied for two interdivision periods after 3 generation of growth.

We varied $E_{collapse}$ and $F_{ext}$ (Figure 5B) to understand how $E_{collapse}$ influences the ability to sustain high $F_{ext}$. We observe three main areas in the resulting phase diagram of aspect ratio (Figures 5C and 5D). Low $F_{ext}$ and $E_{collapse}$ (purple zone) generates clumps, consistent with Figure 4. In contrast, microcolonies with high $E_{collapse}$ maintain high aspect ratio cord structures without breaking for low to moderate values of $F_{ext}$ (orange zone). High $F_{ext}$ disrupts microcolony structures and lowers aspect ratios, although higher $E_{collapse}$ enables microcolonies to sustain a higher $F_{ext}$ without breaking (dark blue zone) (Figures 5B–5E). We define the structural integrity of a cord as the ability to maintain a high aspect ratio despite the presence of $F_{ext}$. We quantified the maximum force $F_{\max}$ that a cord can sustain while maintaining an aspect ratio above an arbitrary threshold of 4 (Figures 5E and 5F). Intuitively, the model predicts that $F_{\max}$ increases as the $E_{collapse}$ increases, e.g., $E_{collapse}=2.7$ a.u, for $F_{\max}=0.8$ a.u., while $F_{\max}=0.3$a.u. disrupts microcolonies with $E_{collapse}=0.3$ a.u. (Figures 5E and 5F), in agreement with Figure 5B.

The mechanical rigidity of cords arises from energy storage in the mycomembrane arising from interbacterial interactions, a picture consistent with the worm-like bundle model (Figure S5B). Together, this enables Mtb—a slow-growing, non-flagellated bacterium with high $E_{collapse}$—to exert force on surrounding cells and spread into new tissue microenvironments via collective effects.

### Mtb cords harbor antibiotic-tolerant bacteria

**Figure S6:**
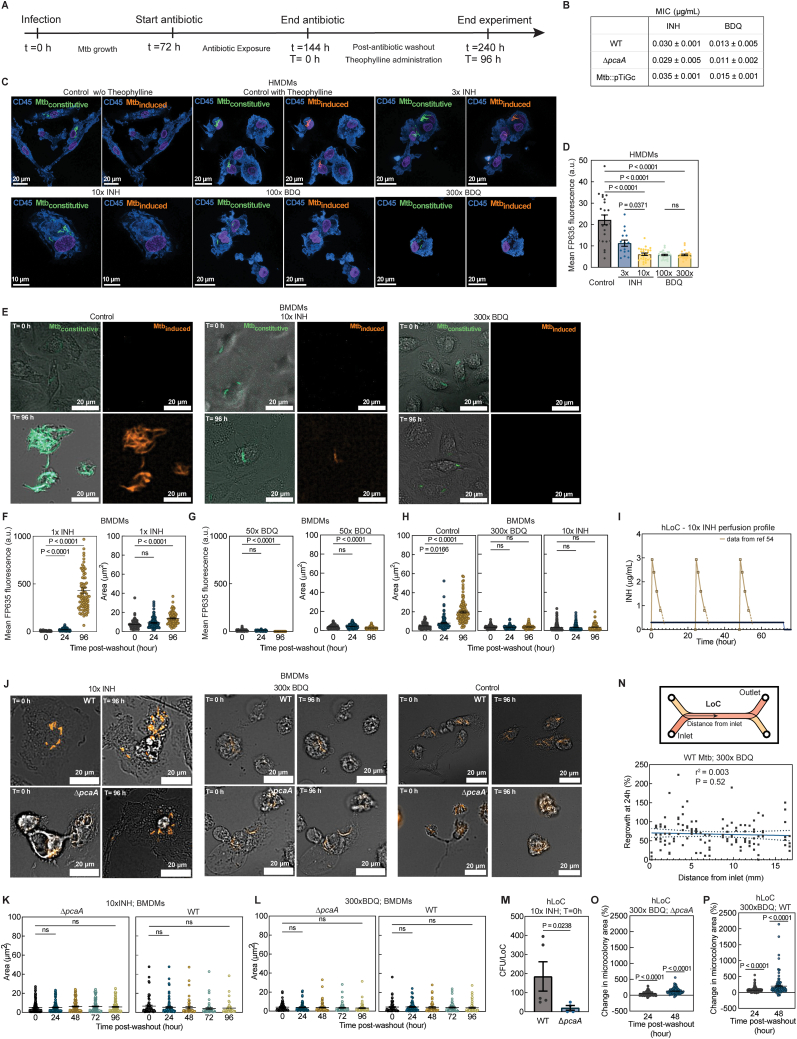
Mtb response to antibiotic therapy, related to Figure 6 (A) Timeline of Mtb infection, antibiotic treatment, and washout in all experiments. (B) Axenic culture MICs. (C and D) Representative confocal images (C) and mean FP635 fluorescence (D) of Mtb::pTiGc-infected, antibiotic-treated HMDMs at T = 24 h (n > 16). Constitutive GFP (green), inducible FP635 (amber), and CD45 (blue). (E) Representative wide-field images of Mtb::pTiGc-infected, antibiotic-treated BMDMs at T = 0 and 96 h. (F and G) Mean FP635 fluorescence and Mtb::pTiGc microcolony area at T = 0, 24, and 96 h in 1× INH (F) and 50× BDQ-treated BMDMs (G) (n > 70 foci of infection). (H) Mtb::pTiGc microcolony area in antibiotic-treated BMDMs at T= 0, 24, and 96 h (n > 90 foci of infection). (I) AUC of serum INH^54^ and for 10× INH perfusion in human LoCs. (J) Representative wide-field images of WT- and Δ*pcaA*-infected, antibiotic-treated BMDMs at T = 0 and 96 h. Bacteria (amber). (K and L) Δ*pcaA* and WT microcolony area in 10× INH- (K) and 300× BDQ-treated BMDMs (L) at T = 0–96 h (n > 80 foci of infection). (M) CFU from WT- and Δ*pcaA*-infected human LoCs treated with 10× INH at T = 0 h (n > 4). (N–P) Change in bacterial microcolony area over T = 24 h (N) and T = 24–48 h relative to T = 0 h in (O) Δ*pcaA*- and (N and P) WT-infected human LoCs and treated with 300× BDQ (n > 80 foci of infection from n = 2 LoCs, n = 26 spatial locations in N).

**Figure 6:**
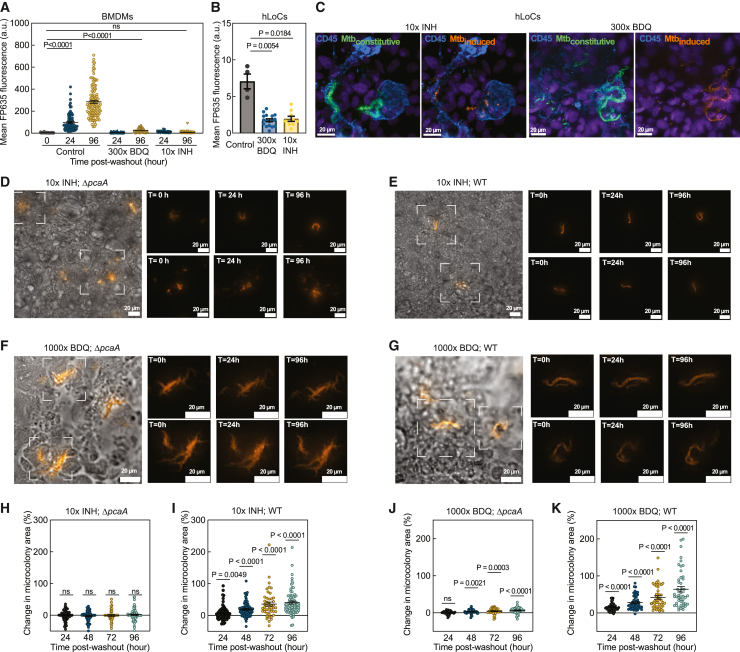
Mtb cords harbor antibiotic-tolerant bacteria (A) Mean FP635 fluorescence of Mtb::pTiGc in antibiotic-treated BMDMs (n > 65) after antibiotic washout. (B) Mean FP635 fluorescence of Mtb::pTiGc in antibiotic-treated human LoCs (n > 5 from n = 2 LoCs) at T = 24 h. (C) Representative confocal images of Mtb::pTiGc-infected human LoCs treated with antibiotics at T = 24 h. Constitutive GFP (green), inducible FP635 (amber), and CD45 (blue). (D–G) Representative time-lapse microscopy snapshots of (D and F) Δ*pcaA*-infected and (E and G) WT-infected human LoCs (E and G) treated with 10× INH (D and E) and 1,000× BDQ (F and G) at T = 0, 24, and 96 h. Bacteria (amber). (H–K) Change in bacterial microcolony area over T = 24–96 h relative to T = 0 h in Δ*pcaA*-infected (H and J) and WT-infected (I and K) human LoCs treated with 10× INH (H and I, n > 71 foci of infection from n = 3 LoCs) and 1,000× BDQ (J and K, n > 43 foci of infection from n = 2 LoCs). Scale bars, 20 μm. See also Figure S6.

The tight packing of bacteria within cords could result in heterogeneous drug penetration in different regions of the cord. This may enhance the stochastic capability of individual drug-sensitive bacteria to survive drug treatments via altered uptake or efflux of drug. We used the expression of the theophylline-inducible far-red fluorescent protein (turboFP635; hereinafter referred to as FP635) in the Mtb::pTiGc strain to monitor translational activity in individual bacteria after prolonged antibiotic therapy (Figure S6A).^53^ We benchmarked FP635 induction in BMDM and human monocyte-derived macrophage (HMDM) monoculture infections (where cord growth is not prominent) against treatment with the hydrophilic drug isoniazid (INH) and the lipophilic drug bedaquiline (BDQ), which are frontline treatments for drug-sensitive and drug-resistant TB, respectively. Untreated bacteria robustly induced FP635 fluorescence (Figures 6A and S6C), but induction reduced sharply at antibiotic concentrations just above the minimum inhibitory concentration (MIC) determined in axenic culture (Figures S6B–S6D). Exposure of infected macrophages to 10× MIC of INH and 100× and 300× MIC of BDQ completely abrogated FP635 induction in intracellular bacteria (Figures 6A, S6C, and S6D). Time-lapse imaging with constant theophylline exposure (Figure S6E) showed that at near-MIC antibiotic concentrations (Figures S6F and S6G), growth resumption was in all instances presaged by FP635 induction. It also confirmed that at the highest doses of antibiotics tested, neither FP635 induction nor regrowth occurred up to 96 h after cessation of antibiotics (Figure S6H).

Next, we probed Mtb::pTiGc responses to constant vascular perfusion of antibiotics in human LoCs at concentrations that were sterilizing in macrophages. Strikingly, bacteria induced FP635 expression within 24 h after cessation of treatment with both antibiotics (Figures 6B and 6C). 10× INH had a more potent effect against Mtb—only isolated bacteria within cord architectures displayed translational activity 24 h post-washout, whereas several regions at the core of Mtb cords retained translational activity post-300× MIC BDQ treatment (Figure 6C).

Several features of the LoC model better recapitulate drug delivery *in vivo*; the continuous perfusion of 10× INH over 24 h generates an area under the curve (AUC) = 7.2 μg h/mL, comparable with measurements in human serum from patients with pulmonary TB (AUC = 8.05 μg h/mL, 95% confidence interval [CI]: 4.88–11.22 μg h/mL)^54^ (Figure S6I), and BDQ concentrations used are comparable with the maximum concentration (C_max_) recorded in human serum.^55^ Although adsorption to polydimethylsiloxane (PDMS) in the LoC device likely reduces the free concentration of the lipophilic BDQ,^56^ this is unlikely to occur with the hydrophilic INH (STAR Methods). These results demonstrate how the better recapitulation of tissue barriers and microenvironments in the LoC model makes it an effective tool to assess the impact of tissue physiology on drug delivery.

Next, we compared the recovery of WT and Δ*pcaA* strains post-antibiotic washout in all *in vitro* models of infection. Both strains had the same MICs as Mtb::pTiGc (Figure S6B) and did not regrow up to 96 h post-washout when exposed to 10× INH and 300× BDQ in macrophage infections (Figures S6J–S6L). On-chip, Δ*pcaA* microcolonies did not regrow up to 96 h after cessation of treatment with 10× INH (Figures 6D and 6H), whereas slow but significant regrowth occurred in WT microcolonies (Figures 6E and 6I). This correlated with a reduced recovery of viable colony-forming unit (CFU) from Δ*pcaA*-infected LoCs (Figure S6M). Although both strains regrew within 24 h after treatment with 300× BDQ, regrowth was faster in WT microcolonies with no evident spatial gradient in regrowth kinetics along the LoC (Figures S6N–S6P). To mitigate losses due to PDMS adsorption, we further examined the responses of both strains to perfusion with 1,000× BDQ. This confirmed the ability of WT microcolonies to resume growth earlier, albeit with lower growth rates (Figures 6F, 6G, 6J, and 6K). Thus, in addition to the known challenges of antibiotic availability at intracellular niches at airway sites of infection, our results demonstrate that cording architectures diminish the potency of antibiotic therapy.

## Discussion

Our study demonstrates that cord formation is a natural adaptation of Mtb to the alveolar microenvironment and can be observed in two separate mouse models by examination of thicker lung slices. We find that cord architectures play key roles in several characteristic features of Mtb pathogenesis, which we delineate by comparison with a cording-deficient strain that is not attenuated for pulmonary growth and expresses well-known effectors of pathogenesis. The first is the relatively delayed innate immune response to intracellular Mtb by tissue-resident AMs.^18^^,^^57^ The compression of the cellular nucleus by mechanical forces exerted by cords contributes to the overall suppression of immune signaling. Furthermore, reduced HDAC1 activity, a consequence of nuclear compression, may promote anti-oxidative *Nrf2*-driven AM responses to Mtb infection.^57^^,^^58^ Reduced HDAC1 activity could also contribute to a delayed inflammatory response by acting in combination with other mechanisms, including the ability of cords to evade cell-autonomous immunity.^8^ Although this study has focused on the effect of Mtb cord formation on the nucleus, the substantial volume of the cytoplasm that can be occupied by bacteria in cords could also compress other cellular organelles and perturb the activity of other HDAC enzymes with cytoplasmic activity.^59^

The trend toward increased immune responses in infections with cording-deficient strains persists well into the chronic stage of infection. We show that cord architectures definitively contribute to the characteristic biogeography of Mtb infections in the interstitial space, needed for subsequent dissemination via erosion of lesions into bronchi. This likely occurs due to the combination of two mechanisms: first, increased recruitment of immune cells to the alveolar space, thereby reducing AM migration to the interstitium and second, by the intercellular spread of Mtb cords between tight junctions of epithelial cells. It is possible that this latter mechanism, which is distinct from transcytosis of individual bacteria,^60^ also operates in other manifestations of Mtb such as tuberculous meningitis^61^ as well as in recently identified *Mycobacterium abscessus* biofilms in the interstitial tissue of cystic fibrosis patients.^62^ Notably, all of the characteristics identified for cording-deficient strains viz. delayed dissemination to the spleen, elevated neutrophilic inflammation and Th17 responses, and alveolar pneumonia were recently ascribed to clinical Mtb isolates with low transmission potential in both the C3HeB/FeJ^33^^,^^63^ and guinea pig models.^64^ This hints at a crucial role for cord formation in the transmission of TB, which deserves future investigation.

Although TDM localizes exclusively to the mycomembrane, it is not the only component; the mycomembrane consists of several other hydrophobic (e.g., PDIM) and hydrophilic (e.g., glucans and other glycolipids) components^47^ whose biophysical properties^65^ and overall effect on interbacterial adhesion remain to be characterized. Although this limits the predictive power of agent-based simulations, it is highly instructive that the model we developed, which incorporates storage of energy via compression of the lipids in the free outer leaflet of the mycomembrane, can nonetheless generate stable cord architectures that maintain their structural integrity despite external perturbations. These structural roles for Mtb mycomembrane lipids complement more well-studied immunological roles of these lipids per se.

Our results show that unlike conventional biofilms, Mtb cords do not require additional host or Mtb-derived substrates for their formation, although host cell debris or bacterial-derived cellulose^66^ likely surround intercellular cords. This work advances our conceptual understanding of non-surface attached biofilm architectures and their functional consequences for infection,^67^ and the model developed provides a framework for probing the development of tightly self-adhering microbial structures.

The LoC model allows us to directly demonstrate that tightly packed Mtb bacteria in cords regrow after exposure to clinically relevant antibiotic concentrations. This could be due to reduced bioavailability in host niches^68^ containing cords or reduced penetration to individual bacteria within these architectures or both—mechanisms that remain to be elucidated. This detrimental impact of cord formation on outcomes of antibiotic therapy in TB, consistent with recent data from the mouse model,^66^ could serve as a target for shortening therapeutic regimens.

**Figure S7:**
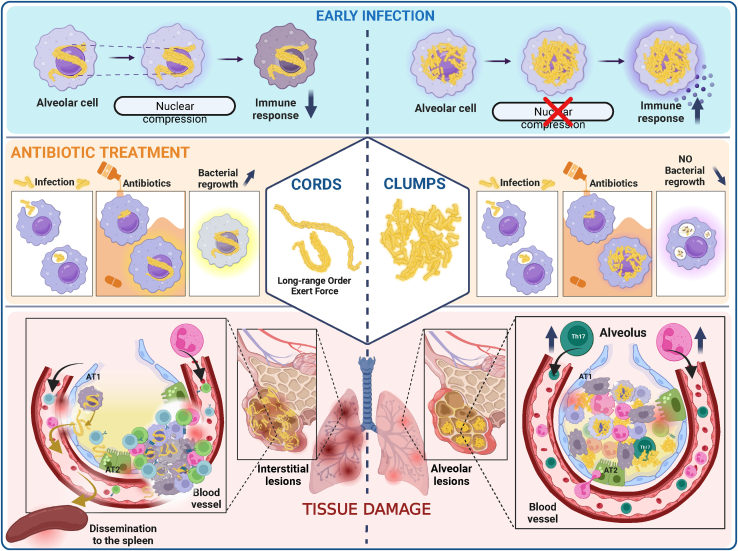
Summary of the mechanopathological effects of Mtb cording, related to Figures 1, 2, 3, 4, and 6

Archetypical schematics of Mtb lesions frequently depict aggregates of several cell types and numerous, individual Mtb bacteria within a subset of these. Our study seeks to amend this scheme by demonstrating the presence of multicellular Mtb architectures from an early stage in pathogenesis and assigning them distinct immunological and physiological roles in pathogenesis with deleterious consequences for antibiotic therapy (Figure S7).

### Limitations of the study

The LoC model reconstitutes several aspects of alveolar biology using primary cells, but the monocyte-derived macrophages used do not yet recreate all the phenotypes of tissue-resident AMs. The osmotic compression induced by PEG is not specific to the nucleus and acts on all host organelles, thereby generating more severe phenotypes than caused by cytosolic growth of Mtb cords. Identification of intercellular cords within lung tissue is challenging even with confocal microscopy, owing to the limits imposed by the optical resolution of the microscope and scattering within the tissue. The experiments to measure lipid compressibility did not quantify the concentration and proportions of different mycolates extracted from the WT and Δ*pcaA*. The agent-based model does not incorporate polar bacterial growth.

## STAR★Methods

### Key resources table

**Table:** 

REAGENT or RESOURCE	SOURCE	IDENTIFIER
**Antibodies**		

CD45 (MEM-28); Anti-human; Mouse monoclonal	Abcam	Cat#: ab8216; RRID: AB_306361
HDAC1 (EPR460(2)); Anti-mouse, anti-human; Rabbit monoclonal	Abcam	Cat#: ab109411; RRID:AB_10861012
HDAC3 (Y415); Anti-mouse, anti-human; Rabbit monoclonal	Abcam	Cat#: ab32369; RRID: AB_732780
CD45 (30-F11); Anti-mouse; AF647 conjugated; Rat monoclonal	BioLegend	Cat#: 103124; RRID: AB_493534
Podoplanin (eBio 8.1.1); Anti-mouse; AF488 conjugated; Syrian Hamster monoclonal	Thermo Fisher Scientific	Cat#: 53-5381-82; RRID: AB_1106990
CD170 (Siglec F) Monoclonal Antibody (1RNM44N), Super Bright™ 436, eBioscience™	Invitrogen	Cat#: 62-1702-82; RRID: AB_2664259
GroEL2 Clone IT-56 (CBA1); anti-Mtb; Mouse Monoclonal	BEI Resources	ATCC No: NR-13655; RRID: SCR_013698
EsxB; anti-Mtb; Rabbit polyclonal antiserum	BEI Resources	ATCC No: NR-13801; RRID: SCR_013698
donkey anti-mouse Alexa Fluor 488	Thermo Fisher	Cat#: A21206; RRID: AB_2535792
donkey anti-mouse Alexa Fluor 647	Thermo Fisher	Cat#: A31573; RRID: AB_2536183
donkey anti-Rabbit Alexa Fluor 488	Thermo Fisher	Cat#: A21206; RRID: AB_2535792
donkey anti-Rabbit Alexa Fluor 647	Thermo Fisher	Cat#: A31573; RRID: AB_2536183
Goat anti-rabbit, HRP-conjugated immunoglobulins, polyclonal	Dako, Denmark	Cat#: P0448; RRID: AB_2617138
Rabbit anti-mouse, HRP-conjugated immunoglobulins, polyclonal	Dako, Denmark	Cat#: P0161; RRID: AB_2687969

**Bacterial and virus strains**		

*Mycobacterium tuberculosis* Erdman strain	John McKinney lab	N/A
Erdman Δ*pcaA*	Michael Glickman lab	PMID: 10882107
Erdman Δ*pcaA attB::pcaA*	Michael Glickman lab	PMID: 10882107
Erdman-pND257-tdTomato	John McKinney lab	PMID: 33228849
Erdman Δ*pcaA*-pND257-tdTomato	This paper	N/A
Erdman-pTiGc-gfp-turboFP635	This paper	N/A

**Biological samples**		

Murine alveolar epithelial cells	Cell Biologics	Cat#: C57-6053
Murine lung microvascular endothelial cells	Cell Biologics	Cat#: C57-6011
Human alveolar epithelial cells	Cell Biologics	Cat#: H-6053
Human lung microvascular endothelial cells	Cell Biologics	Cat#: H-6011
Buffy Coat Blood	Interregionale Blutspende SRK AG	N/A

**Chemicals, peptides, and recombinant proteins**		

Native Collagen, Bovine dermis	AteloCell	Cat#: IAC-50
Fibronectin	Thermofisher	Cat#: 33016015
Gibco Recombinant murine M-CSF	Thermofisher	Cat#: PMC2044
Recombinant human M-CSF	Bio-Techne	Cat#: 216-MCC-010/CF
Cultrex Reduced Growth Factor Basement Membrane Extract, Type 2, PathClear	Bio-Techne	Cat#:3533-005-02
EDTA	Merck	Cat#:E99884
Cell Recovery Solution	Corning	Cat#: 354253
Dexamethasone	Merck	Cat#: 265005-100MG
Difco ™ Middlebrook 7H9 broth	Beckton Dickinson	Cat#: 271310
BBL™ Middlebrook OADC enrichment	Beckton Dickinson	Cat#: 212351
Tyloxapol	Sigma Aldrich	Cat#: T8761-50G
7H11 Agar	Beckton Dickinson	Cat#: 283810
MGIT ™ PANTA ™	Beckton Dickinson	Cat#: 245114
Curosurf ™	Chiesi Pharmaceuticals	N/A
Gibco Glutamax™, 100X	ThermoFischer	Cat#: 35050-038
Gibco HEPES buffer, 1M	ThermoFischer	Cat#: 15630106
Human Primary Epithelial Cell Medium	Cell Biologics	Cat#: H6621
Human Primary Endothelial Cell Medium	Cell Biologics	Cat#: H1168
Human Primary Endothelial Cell Medium phenol red free	Cell Biologics	Cat#: H1168PF
Mouse Primary Epithelial Cell Medium	Cell Biologics	Cat#: M6621
Mouse Primary Endothelial Medium	Cell Biologics	Cat#: M1168
Mouse Primary Endothelial Medium (Phenol Red free)	Cell Biologics	Cat#: M1168PF
Gibco DMEM Medium, Phenol-Red free	Thermofisher	Cat#: 31053-028
Gibco RPMI Medium, Phenol Red free	Thermofisher	Cat#: 11835030
Recombinant Mouse Interferon-gamma	Merck	Cat#: IF005
Polyethylene Glycol	Merck	Cat#: 202398
TRIzol	Thermofisher	Cat#: 15596018
16% Paraformaldehyde solution, methanol free	Thermofisher	Cat#: 28908
25% Glutaraldehyde solution, aqueous	Electron Microscopy Sciences	Cat#: 16210
Durcupan ACM	Electron Microscopy Services	Cat#: 14040
Invitrogen™ Hoechst 34580Na	Thermofisher	Cat#: H21486
TrypLE™ Express Enzyme (1X)	Thermofisher	Cat#: 12605010
Phosphate Buffered Saline	Thermofisher	Cat#: 10010056
Prolong Gold Antifade Mountant	Thermofisher	Cat#: P36930
TSA Opal570	Akoya Biosciences	Cat#: FP1488001KT
TSA Opal650	Akoya Biosciences	Cat#: FP1496001KT
TSA Opal690	Akoya Biosciences	Cat#: FP1497001KT

**Critical commercial assays**		

CD14 Microbeads ultrapure, human	Miltenyi Biotec	Cat#: 130-118-906
Lung Dissociation Kit, Mouse	Miltenyi Biotec	Cat#: 130-095-927
Cytokine Array – Human Cytokine Antibody Array (Membrane, 23 Targets)	Abcam	Cat#: AB133996
RNAscope hs *IL1B* probe	Bio-Techne	Cat#: 310361
RNAscope hs *IFNB1* – C4 probe	Bio-Techne	Cat#: 417071-C4
RNAscope hs *IL6* – C2 probe	Bio-Techne	Cat#: 310371-C2
RNAscope Multiplex Fluorescent V2 assay	Bio-Techne	Cat#: 323110
RNeasy Micro Plus Kit	Qiagen	Cat#: 74034
RNeasy Micro Kit	Qiagen	Cat#: 74004
RNeasy Mini Kit	Qiagen	Cat#: 74104
SuperScript IV First Strand Synthesis System	Invitrogen	Cat#: 18091050
NEBNext® Ultra™ II RNA Library Prep Kit	New England Biolabs	Cat#: E7770S

**Deposited data**		

Code for Agent-based model, Langmuir monolayer analysis, raw data for Figures	Zenodo	https://doi.org/10.5281/zenodo.7740873
RNA-seq analysis	Geo	GSE238102

**Experimental models: cell lines**		

L-929 NCTC clone 929	ATCC	Cat#: CCL-1™, RRID:CVCL_0462

**Experimental models: organisms/strains**		

C3HeB/FeJ Mouse	Jackson Laboratory	RRID: IMSR_JAX:000658
C57BL/6 Mouse	Charles River Laboratory	RRID: IMSR_JAX:000664
C57BL/6-Tg(CAG-EGFP)131Osb/LeySopJ Mouse	Jackson Laboratory	RRID: IMSR_JAX:006567

**Oligonucleotides**		

GGT GCC TAT GTC TCA GCC TCT T	Microsynth	mm_*Tnfa*_fwd
GCC ATA GAA CTG ATG AGA GGG AG	Microsynth	mm_*Tnfa*_rev
TGG ACC TTC CAG GAT GAG GAC A	Microsynth	mm_*Il1b*_fwd
GTT CAT CTC GGA GCC TGT AGT G	Microsynth	mm_*Il1b*_rev
TAC CAC TTC ACA AGT CGG AGG C	Microsynth	mm_*Il6*_fwd
CTG CAA GTG CAT CAT CGT TGT TC	Microsynth	mm_*Il6*_rev
GCT ACA AGA GGA TCA CCA GCA G	Microsynth	mm_*Ccl2*_fwd
GTC TGG ACC CAT TCC TTC TTG G	Microsynth	mm_*Ccl2*_rev
CAGAAGGATCACCAGTAGTCGG	Microsynth	mm_*Ccl7*_fwd
ATAGCCTCCTCGACCCACTTCT	Microsynth	mm_*Ccl7*_rev
GGGTGCTGAAAAGCTACGAGAG	Microsynth	mm_*Ccl8*_fwd
GGATCTCCATGTACTCACTGACC	Microsynth	mm_*Ccl8*_rev
TCCAGAGCTTGAAGGTGTTGCC	Microsynth	mm_*Cxcl1*_fwd
AACCAAGGGAGCTTCAGGGTCA	Microsynth	mm_*Cxcl1*_rev
CAT CCA GAG CTT GAG TGT GAC G	Microsynth	mm_*Cxcl2*_fwd
TAC CAC TTC ACA AGT CGG AGG C	Microsynth	mm_*Cxcl2*_rev
TGAAGCCTCACCGAATCCGCAT	Microsynth	mm_*Hdac1*_fwd
TGGTCATCTCCTCAGCATTGGC	Microsynth	mm_*Hdac1*_rev
CAGACTACCTCAACCGTTCCAC	Microsynth	mm_*Il17a*_fwd
TCCAGCTTTCCCTCCGCATTGA	Microsynth	mm_*Il17a*_rev
CAGCAACAGCAAGGCGAAAAAGG	Microsynth	mm_*Ifng*_fwd
TTTCCGCTTCCTGAGGCTGGAT	Microsynth	mm_*Ifng*_rev
CGTGCAAGCTCATTACGACC	Microsynth	Mtb*_pcaA_*fwd
CGTCGTATTTCTCGATGGCG	Microsynth	Mtb*_pcaA_*rev
CGAAGAGTCGCAATCGATTTAC	Microsynth	Mtb*_umaA_*fwd
CGTAGTTCTCGATCGCTCGTT	Microsynth	Mtb*_umaA_*rev
ACTACGACCTGTCCGACG	Microsynth	Mtb*_mmaA2_*fwd
CGTTGACGTCATACTGCGC	Microsynth	Mtb*_mmaA2_*rev
CTTCCATGACAATCCAGCTGC	Microsynth	Mtb*_papA5_*fwd
GATGCAATAGGGACACGCTC	Microsynth	Mtb*_papA5_*rev
GCAAAAAACATAGTGAACGCCAGA	Microsynth	Mtb*_fadD26*_fwd
TCCGGGTAACCCGCATAA	Microsynth	Mtb*_fadD26_*rev

**Recombinant DNA**		

pND-257	PMID: 33228849	N/A
pTiGc	PMID: 27027532	Addgene (#78314)

**Software and algorithms**		

Imaris 9.9	Bitplane	RRID:SCR_007370
Huygens Software 2.7.8	SVI	RRID:SCR_014237
Prism 9.5.1	Graphpad	RRID:SCR_002798
ImageJ	NIH	RRID:SCR_003070
TrakEM2	Albert Cardona	RRID:SCR_008954
Blender 2.7.4	Blender Foundation	RRID:SCR_008606
MATLAB 2018b	MathWorks	RRID:SCR_001622
Molecular Signatures Database	UC San Diego, Broad Institute	RRID:SCR_016863
Gene Set Enrichment Analysis (GSEA)	UC San Diego, Broad Institute	RRID:SCR_003199
R Project for Scientific Computing	Free Software Foundation	RRID:SCR_001905
Star	PMID: 23104886	RRID:SCR_004463
FastQC	Baraham Institute	RRID:SCR_014583
Bioconductor	Roswell Park Comprehensive Cancer Center	RRID:SCR_006442

**Other**		

NalgeneTM square PETG media bottles with closure	ThermoFisher	Cat#: 2019-0030
Lung-on-chip	Emulate	Cat#: Chip-S1
Chip Coating Reagents	Emulate	Cat#: ER1, ER2
1.30/1.00 x 15 mm metallic tubes	Unimed	Cat#: 200.010-A
1.30 x 10 mm metallic tubes	Unimed	Cat#: 200.010-A
1.30 x 10 mm solid metallic tubes	Unimed	Cat#: 200.020-A
PharmaMed tubing ID = 0.035 inches OD = 0.101 inches Wall =0.034 inches	Cole-Parmer	Cat#:95809-26
5-μm syringe filter	Millipore	Cat#: SLSV025LS
BioGen Pro 200 Homogenizer	Pro Scientific	Cat#: 01-01200
gentleMACS TM Octo Dissociator with Heaters	Miltenyi Biosciences	Cat#: 130-096-427
LS Columns	Miltenyi Biosciences	Cat#: 130-042-401
MACS Multistand	Miltenyi Biosciences	Cat#: 130-042-303
Precellys 24 Tissue Homogenizer	Bertin Instruments	Cat#: P000669-PR240-A
Langmuir-Blodgett balance	Nima Technologies	N/A
24 x 60 thickness 1 coverslip holder - magnetic	Okolab	Cat#: 1x24x60-M
35mm holder - magnetic	Okolab	Cat#:1x35-M
μ-Dish 35 mm, high	Ibidi	Cat#:81156
Aladdin programmable syringe pump	WPI	Cat#:PUMP-NE-1000
Fusion 100-X syringe pump	Chemyx	Cat#: 0710X
Eclipse Ti-2 inverted microscope	Nikon	RRID:SCR_021068
Thunder Imager Live Cell and 3D assay	Leica	RRID: SCR_023794
Leica SP8 confocal microscope	Leica	RRID:SCR_018169
3view system SEM	Gatan	RRID:SCR_019929

### Resource availability

#### Lead contact

Further information and requests for resources and reagents should be directed to and will be fulfilled by the lead contact, Vivek V. Thacker (vivek.thacker@med.uni-heidelberg.de).

#### Materials availability

The Δ*pcaA*::pND257-tdTomato Mtb strain generated for this study is available from the lead contact upon reasonable request.

### Experimental model and study participant details

#### Animal Models

C57BL/6 mice (RRID: IMSR_JAX:000664) were purchased from a commercial supplier (Charles River). C3HeB/FeJ mice (RRID: IMSR_JAX:000658) were bred in-house at EPFL. Female C57BL/6 aged 8 weeks and mixed male and female C3HeB/FeJ mice aged between 8 and 12 weeks were housed in a specific pathogen-free facility. Animal protocols were reviewed and approved by EPFL’s Chief Veterinarian, by the Service de la Consommation et des Affaires Vétérinaires of the Canton of Vaud, and by the Swiss Office Vétérinaire Fédéral under license VD3472 and VD3805. Littermates of the same sex were randomly assigned to experimental groups.

#### Primary Cell culture

Primary human alveolar epithelial cells (ATs) and lung microvascular endothelial cells were obtained from Cell Biologics, USA from anonymized donors and authenticated by the supplier. The sex of the cells was not determined. Both cell types were cultured *in vitro* in complete medium comprising base medium and supplements (Cell Biologics, USA) in 5% CO_2_ at 37°C. All human chips were reconstituted with ATs seeded directly on the LoC, without any additional *in vitro* culture. Endothelial cells were passaged between 3-5 times before seeding in the LoC devices. Experiments were performed with cells from at least two donors.

Peripheral blood mononuclear cells (PBMCs) were obtained from buffy coat (Interregional Blood Transfusion SRC Ltd, Switzerland) taken from anonymized donors. PBMCs were isolated using a Biocol Separation procedure as per the manufacturer’s instructions. Isolated PBMCs were subsequently cryopreserved in a solution of 70% heat-inactivated fetal bovine serum (FBS, Gibco), 20% RPMI medium (Gibco) and 10% dimethyl sulfoxide (DMSO). One week prior to seeding the macrophages in the LoC devices, a cryopreserved aliquot was thawed and cultured in a T-75 flask (TPP, Switzerland) in RPMI supplemented with 10% FBS. CD14+ monocytes were isolated using positive selection (CD14 Ultrapure Isolation Kit, Miltenyi Biosciences), embedded in hemispherical domes of basement membrane extract (BME-2, Cultrex) in 24 well plates, and cultured in RPMI medium supplemented with 10% FBS and differentiated for 7 days with 20 ng/mL recombinant human macrophage-colony stimulating factor protein (M-CSF) (Thermo Fisher Scientific). The monocytes were differentiated for 7 days into human monocyte-derived macrophages (HMDMs), which were subsequently either directly infected with Mtb or added to the epithelial face of the human LoC model.

For murine bone marrow-derived macrophages (BMDMs), bone marrow was obtained from the femurs of 6- to 8-week-old female C57BL/6 mice (Charles River) or the C57BL/6-Tg (CAG-EGFP)131Osb/LeySopJ (Jackson Labs, RRID: IMSR_JAX:006567) mice that constitutively express GFP in every cell and cryopreserved. Animal protocols were reviewed and approved by EPFL’s Chief Veterinarian, by the Service de la Consommation et des Affaires Vétérinaires of the Canton of Vaud, and by the Swiss Office Vétérinaire Fédéral under license VD 3434. Bone marrow was cultured in Dulbecco’s Modified Eagle Medium (DMEM) (Gibco) supplemented with 10% FBS and 20% L929-cell-conditioned medium (as a source for macrophage-colony stimulating factor protein or M-CSF) for 7 days. No antibiotics were used in the cell culture media for all cell types to avoid activation of macrophages or inhibition of Mtb growth.

All primary cells used without passage were verified mycoplasma free by the supplier. Passaged cells and cell culture media were verified mycoplasma free at EPFL.

#### *Mycobacterium tuberculosis* culture

All bacterial strains were derived from Mtb strain Erdman and cultured at 37°C in Middlebrook 7H9 base (Difco) supplemented with 0.5% albumin, 0.2% glucose, 0.085% NaCl, 0.5% glycerol, and 0.02% tyloxapol for liquid culture (hereinafter referred to as 7H9 medium) in 30 mL inkwell bottles or Middlebrook 7H11 (Difco) supplemented with 10% OADC enrichment (BD) and 0.5% glycerol for solid culture unless otherwise stated. Aliquots were stored in 15% glycerol at -80°C and used once. The Erdman Δ*pcaA* strain and the Erdman Δ*pcaA attB::pcaA* strain were kind gifts from the lab of Prof. Michael Glickman at the Memorial Sloan Kettering Cancer Center, USA. The WT Erdman and Erdman Δ*pcaA* strains were transformed with a plasmid (pND257) integrated at the chromosomal attB site to allow constitutive expression of the fluorescent protein tdTomato. The WT Erdman was also transformed with an episomal plasmid expressing green fluorescent protein under the control of the *hsp*60 promoter and/or the inducible far-red fluorescent protein turboFP635 under the control of the *hsp60* promoter engineered to contain a riboswitch that binds to theophylline (Mtb::pTiGc).^53^

### Method details

#### Characterization of axenic culture Mtb aggregates

Exponential phase cultures of WT, Δ*pcaA* and the Erdman Δ*pcaA attB::pcaA* Mtb strains in 7H9 were centrifuged at 200 g for 3 minutes to precipitate large clumps. The supernatant consisting of single cells or small aggregates was centrifuged again at 4500 g for 5 minutes, the supernatant discarded, the pellet resuspended in 6 mL of 7H9 medium without the addition of Tyloxapol and cultured in 30 mL inkwells. The cultures were then examined using live-cell wide-field microscopy 3-4 days later to visualize Mtb cords.

#### Murine and human LoC models

The protocols for both murine^19^ and human^20^ LoCs have been previously reported. In brief, devices made of polydimethylsiloxane (PDMS) were obtained from Emulate (Boston, USA). Chips were activated using ER-1 solution (Emulate) dissolved in ER-2 solution at 0.5 mg/mL (Emulate) and exposed twice for 20 minutes under UV light. Chips were then washed thoroughly with PBS before incubating with an ECM solution of 150 μg/mL bovine collagen type I (AteloCell, Japan) and 30 μg/mL fibronectin from human plasma (Sigma-Aldrich) in PBS buffered with 15 mM HEPES solution (Gibco) for 1-2 hours at 37°C. If not used directly, coated chips were stored at 4°C and pre-activated before use by incubation for 30 minutes with the same ECM solution at 37°C.

Primary murine or human pulmonary microvascular endothelial cells between passages four and eight were cultured overnight at 37°C and 5% CO_2_ in T-75 cell culture flasks, detached with 0.05% Trypsin, concentrated to 5-10 million cells/mL, and seeded on the bottom face of the PDMS membrane. The chip was then incubated for a short period at 37°C to allow the endothelial cells to spread and subsequently seeded with ATs. For human LoCs and surfactant-competent (NS) murine LoCs, freshly isolated human and murine ATs were seeded directly from cryopreserved vials received from the supplier, each vial of 0.5 million cells could be used to seed between two and three murine LoCs or up to four human LOCs. For murine surfactant-deficient (DS) LoCs, ATs were passaged up to passage eight, cultured overnight at 37°C and 5% CO_2_ and seeded at 1-2 million cells/mL. The chip was incubated overnight with complete endothelial and epithelial media in the respective channels under static conditions. The following day, the chip was washed and a reduced medium for the air-liquid interface (ALI) was flowed through the vascular channel using syringe pumps (F100X Precision Syringe Pumps, Chemyx Inc.) at 60 μL/hour. The composition of the ALI media used was as previously described but with an FBS concentration of 5% (murine LoCs) or 2% (human LoCs). The epithelial face was incubated with epithelial base medium with 200 nM (murine LoCs) or 1 μM (human LoCs) dexamethasone (Sigma-Aldrich) without FBS supplementation to promote tight junction formation and surfactant expression as reported in previous LoC studies. Flow was maintained over 2 days with daily replacement of the medium on the epithelial face (with dexamethasone supplementation). At the end of this period, for murine LoCs for living imaging, GFP-expressing BMDMs differentiated for 7 days in murine M-CSF were detached from the petri dish using 2 mM ethylenediaminetetraacetic acid (EDTA, Sigma-Aldrich) in PBS at 4°C, centrifuged at 200 g for 5 minutes, resuspended in a small volume of epithelial cell media without dexamethasone, and introduced onto the epithelial face. The chip was incubated for 30 minutes at 37°C and 5% CO_2_ to allow macrophages to attach to the ATs. For human LoCs, BME-2 hydrogel domes containing CD14+ monocytes differentiated for 7 days in human M-CSF into macrophages were detached by mechanical scraping and brought to a semi-liquid state by adding 500 μL of ice-cold RPMI medium per well. The BME-RPMI suspension was then centrifuged at 200 g for 5 minutes in a 15 mL tube pre-coated with 1% BSA in PBS, resuspended in 4-5 mL of ice-cold Cell Recovery Solution (Corning) per well, and incubated at 4°C for 30 minutes. The cell suspension was then washed with 10 mL RPMI medium supplemented with 10% FBS to remove any remaining traces of the recovery reagent. Isolated macrophages were resuspended in a small volume of epithelial cell media without dexamethasone and introduced onto the epithelial face. The chip was incubated for 30 minutes at 37°C and 5% CO_2_ to allow macrophages to attach to the ATs. In both cases, the medium on the epithelial face was then removed, and the chip was maintained overnight at an air-liquid interface. Chips were controlled to ensure that they successfully maintained the ALI overnight and then transferred to the biosafety level 3 (BSL-3) facility for Mtb infection. No antibiotics were used in any of the cell culture or flow media for infection experiments.

#### Infection of LoCs with Mtb

LoCs were assembled into a stage top incubator (Okolab) prior to infection and flow of medium through the vascular channel was maintained throughout the course of the experiment by use of a syringe pump. A 1 mL aliquot of an Mtb culture grown to exponential phase (OD_600_ 0.3-0.5) was centrifuged at 5000 g for 5 minutes at room temperature, the supernatant was removed, and the cell pellet was resuspended in 200 μL of epithelial cell media without FBS. A single cell suspension was generated via filtration through a 5-μm syringe filter (Millipore). The single-cell suspension was diluted 100-fold in epithelial media and 30 μL was added to the epithelial channel of the LoC. The infectious dose was measured by plating serial dilutions of the single-cell suspension on 7H11 plates and counting CFU after 3-4 weeks of incubation at 37°C and varied between 200 and 800 Mtb bacilli. The chip was incubated for 2-3 hours at 37°C and 5% CO_2_ to allow Mtb infection of cells on the epithelial face, after which the solution on the epithelial face was withdrawn. The epithelial face was returned to ALI and the inlets of the infected chip were sealed with solid pins as a safety precaution for incubation and imaging in the BSL-3 facility. The proportion of bacteria that remained on the chip was estimated by plating serial dilutions of the withdrawn solution on 7H11 plates and counting CFUs after 3-4 weeks of incubation at 37°C.

For antibiotic treatment and regrowth experiments on chip, antibiotic stocks were prepared at 10 mg/mL in DMSO and frozen at -20°C. At 3 dpi, antibiotic stocks were thawed, serially diluted to the appropriate concentrations in pre-warmed ALI medium and loaded on fresh 10 mL syringes to setup flow on the endothelial side using syringe pumps. After 3 days of continuous antibiotic perfusion, LoCs were switched back to perfusion with pre-warmed ALI media (with 1 mM theophylline for experiments with Mtb::pTiGc only) in fresh syringes to allow antibiotic washout for up to 4 subsequent days. At specific timepoints after washout, LoCs were either processed for CFU assays, or fixed with PFA for confocal imaging or used for live-cell wide-field imaging as required.

To obtain CFU from infected LoCs after antibiotic treatment, the apical and vascular channels were incubated for ca. 10 minutes with a solution of 0.05% sodium dodecyl sulphate (SDS) in PBS, and subsequently washed with this solution until a visual inspection by brightfield microscopy revealed the majority of cells on the LoC were disrupted and extracted. Thereafter, the vascular and apical channels were washed rapidly with a solution of RLT buffer diluted two-fold in PBS to extract all remaining mycobacteria. Solutions from all washout steps per LoC were collected in a 10-fold excess of 7H9 medium and centrifuged at 4500g for 10 minutes to concentrate the bacterial population in the solution. Thereafter, supernatant was discarded, the pellet was resuspended in ca. 500 μL of 7H9 medium and then used for CFU assays on 7H11 agar plates.

#### Infection of BMDMs, HMDMs and ATs with Mtb

BMDMs or HMDMs were seeded in standard 6 or 12-well plates (for experiments for endpoint RNA collection) or 24-well plates (Ibidi) suitable for high-resolution imaging. Murine ATs were grown to confluence in T-75 tissue culture flasks, and then seeded into 35 mm Ibidi cell culture dishes and cultured in murine epithelial cell media supplemented with 10% FBS until a confluent monolayer was obtained. Mtb strains were grown to mid-exponential phase and 1 mL aliquots of the cultures were centrifuged at 5000 g for 5 minutes, the pellet resuspended in 200 μL of BMDM/ HMDM culture medium and passed through a 5 μm syringe filter to obtain a single cell suspension. The single cell suspension was diluted ca. 100-fold in BMDM/HMDM culture medium and 250 μL of the inoculum was added per well to macrophages in a 12 or 24 well plate. After 4 hours of infection, macrophages or epithelial cells were washed extensively with pre-warmed PBS to remove extracellular bacteria and incubated with the appropriate culture medium for indicated durations at 37°C and 5% CO_2_. For experiments involving BMDM activation, BMDMs were pre-treated with 100 U/mL murine interferon gamma (IFN-γ) for 24 hours before infection and maintained in culture medium supplemented with IFN-γ through the course of the experiment. For *in vitro* nuclear compression experiments, polyethylene glycol (PEG) (Sigma) was added at 1:10 v/v to culture medium after 4 hours of infection and washing of extracellular bacteria and maintained through the course of the experiment.

For antibiotic treatment and regrowth experiments in infected BMDMs or HMDMs, antibiotic stocks were prepared at 10 mg/mL in DMSO and frozen at -20°C. At 3 dpi, antibiotic stocks were thawed, serially diluted to the appropriate concentrations in pre-warmed BMDM/HMDM culture medium and added to wells with infected macrophages. After 3 days of continuous antibiotic exposure, media from each well was removed, the macrophages were washed twice with fresh, pre-warmed BMDMs culture medium and replaced with the same for further incubation (with or without theophylline, depending on the experimental strain used). Infected macrophages were then fixed for confocal imaging or used for wide-field imaging as required.

#### Analysis of PDIM production by Mtb strains

Phthiocerol dimycocerosates (PDIM) synthesis was evaluated by thin-layer chromatography (TLC) of bacterial lipids. 10 mL of exponential phase bacteria were labeled with 2 μCi of [^14^C]-propionate (Campro Scientific) for 48 hours. Lipids were extracted from the pellets of the radiolabelled culture using 5 mL of methanol:0.3% NaCl (10:1) and 5 mL of petroleum ether. The mixture was vortexed for 3 minutes, centrifuged at 930 g for 10 minutes, and the upper layer was collected. A second extraction on the lower phase was carried out with 5 mL petroleum ether. The combined extracts were inactivated with an equal volume of chloroform for 1 hour and concentrated through evaporation. The samples were spotted on a 5 x 10 cm TLC silica gel 60 F254 (Merck) and developed in petroleum ether: diethyl ether (9:1). The developed TLC plate was exposed to an Amersham Hyperfilm ECL (GE Healthcare) for chemiluminescence analysis and visualized with a Typhoon Scanner (GE Lifesciences).

#### Extraction of Mtb surface lipids

Mtb strains were inoculated in 10 mL cultures in Sauton’s medium supplemented with 0.05% Tween 80 at a starting OD_600_ of 0.1 and incubated at 37°C with shaking. Thereafter, lipid extraction was performed using petroleum ether: methanol as described above for PDIM extraction, with the lipids collected in glass vials to prevent contamination in plastic Falcon tubes. For Langmuir Blodgett measurements, the lipids were then dried under nitrogen, resuspended in chloroform, and stored at -80°C until further analysis. For experiments where BMDMs were exposed to Mtb surface lipids, the lipids were resuspended in 5 mL chloroform and stored at -20°C until further use.

#### Exposure of BMDMs to LPS

BMDMs differentiated from bone marrow of C57BL/6 mice were reseeded into 6-well plates (Greiner BioOne) and then exposed to lipopolysaccharide (LPS) from *Escherichia coli* (Sigma) in BMDM medium at a concentration of 100 ng/mL for 12 hours. The BMDMs were subsequently processed for RNA extraction or fixed with 4% PFA for immunostaining assays.

#### Exposure of BMDMs to Mtb surface lipid monolayers

Individual wells in a 6-well plate were coated with a solution of 20 μL of surface lipids of WT and Δ*pcaA* in chloroform as extracted and further diluted in 180 μL chloroform. The solution was allowed to uniformly coat the surface of the well plate and evaporate. Thereafter, BMDMs differentiated from bone marrow of C57BL/6 mice were added to the coated wells of the 6-well plate and cultured overnight in BMDM medium. The BMDMs were subsequently processed for RNA extraction.

#### Wide-field imaging and analysis

LoCs or well plates with infected macrophages were placed in a microscope stage-top incubator and mounted on the stage of a wide-field Leica Thunder or Nikon Ti-2 microscope. The stage-top incubator was connected to a gas mixer (Okolab) to maintain 5% CO_2_ and a temperature of 37°C throughout the imaging period. For LoCs, flow of medium through the vascular channel was maintained throughout this period via the use of a syringe pump. The LoC was imaged either using a 25x water immersion objective (NA = 0.95, Leica) for snapshot imaging for data in Figure 6 or a long working distance 20x phase-contrast objective (NA = 0.75, Ph2, Nikon) for long-term time-lapse imaging (imaging interval of 1.5 hours) for data in Figures 1, 3, and S1. The epithelial face of the LoC (where the refractive index differences is highest due to the ALI) was maintained in focus. At each timepoint, a Z-stack of 9-10 images with an axial spacing of 2 μm (Leica) or 10 μm (Nikon) was taken for a series of fields of view along the entire length of the LoC to account for the dynamic 3D movement of the macrophages between both faces, as well as drift in focus over time. Phase-contrast (Nikon) or bright-field (Leica) images were also captured; for live imaging experiments, bacteria that did not co-localize with GFP-expressing macrophages over time were assumed to infect ATs, which was verified by subsequent immunostaining. For data in Figure S1, the growth rate was calculated by measuring the total fluorescence of the microcolony at each timepoint as previously described.^19^ A threshold of growth rate <1/168 h^-1^ was used to identify microcolonies that were non-growing.

For live imaging of infected macrophages, images were acquired with a 20x air objective (Leica) at intervals of 2 hours. Bacteria were identified by the constitutive fluorescent marker (GFP or tdTomato, depending on the strain used). All images were captured with an EMCCD camera (iXON Ultra 888, Andor, Nikon) cooled to -65°C.

#### Animal infection with Mtb

C57BL/6 mice were purchased from a commercial supplier (Charles River). C3HeB/FeJ mice were bred in-house at EPFL. Female C57Bl/6 aged 8 weeks and mixed male and female C3HeB/FeJ mice aged between 8 and 12 weeks were housed in a specific pathogen-free facility. Animal protocols were reviewed and approved by EPFL’s Chief Veterinarian, by the Service de la Consommation et des Affaires Vétérinaires of the Canton of Vaud, and by the Swiss Office Vétérinaire Fédéral under license VD3472 and VD3805. Mice were infected by the aerosol route using a custom-built aerosol machine, as previously described.^69^ Briefly, bacteria were grown to the exponential phase, collected by low-speed centrifugation at 500 rpm for 10 minutes to obtain a single-cell suspension and adjusted to an optical density at 600 nm (OD_600_) of 0.5 in 20 mL PBS supplemented with 0.05% tyloxapol. At 1 dpi, a group of four mice were euthanized by CO_2_ overdose; the lungs were removed aseptically and homogenized in 3 mL of 7H9 medium and plated on 7H11 plates containing the MGIT™ PANTA™ antibiotic mix (BD). Colonies were counted after 4-5 weeks of incubation at 37°C to determine the initial bacterial load from infection.

At designated timepoints, 4-6 mice per experimental group were euthanized by CO_2_ overdose. The lungs were removed aseptically, weighed, and then used for determination of one or more experimental read-outs. For imaging of thick infected tissue sections, ∼ 50 mg of lung tissue was immersed in 20 mL 4% neutral-buffered paraformaldehyde (PFA) solution (Biosystems) and incubated for 2 hours at room temperature followed by 24 hours at 4°C for fixation. The tissue section was then equilibrated in 30% sucrose for a subsequent 24-48 hours and cryofrozen for sectioning. For RNA isolation, ∼ 30 mg of lung tissue was immersed in 1 mL TRIzol (Ambion). The remaining tissue was weighed again and homogenized in 3 mL of PBS, serially diluted, and plated on 7H11 plates with MGIT™ PANTA™ for recovering colony-forming units (CFUs). The plates were incubated at 37°C for up to 5 weeks to obtain CFU counts, which were then normalized to total lung weight to calculate bacterial CFU burden per lung. At designated timepoints, spleens were also removed aseptically from mice and homogenized in 3 mL of PBS and plated on 7H11 plates with MGIT™ PANTA™ for recovering CFUs.

#### Immunofluorescence - macrophages and LoCs

For immunofluorescence labelling, infected macrophages or LoCs were fixed by immersing completely in a freshly diluted solution of 4% paraformaldehyde (PFA) in PBS for 2 hours at room temperature followed by two washes with PBS. The fixed sample was permeabilized with 0.1% Triton X-100 (Sigma-Aldrich) and 2% saponin (Sigma-Aldrich) in PBS for 15 minutes at room temperature followed by incubation with a blocking solution of 2% bovine serum albumin (BSA) in PBS (“blocking buffer”). The permeabilized and blocked sample was then incubated overnight with the primary antibody in the blocking buffer at 4°C. A list of primary and secondary antibodies and concentrations used is included in the key resources table. The following day, the sample was washed extensively with fresh blocking buffer before incubation with the secondary antibody at a dilution of 1:300 for 1 hour at room temperature. Infected macrophages or LoCs were also stained with the Hoechst dye (Thermo Fisher) at 1:1000 dilution for 30 minutes at room temperature for visualizing nuclei. Confocal images were acquired on an inverted Leica SP8 microscope at the EPFL BIOP core facility.

#### Immunofluorescence - lung tissue sections

150 μm thick sections of fixed frozen infected animal tissues were cut using a cryostat (Leica CM3050S). After a blocking step of 2 hours in 1% BSA with 1% Triton X-100 in PBS, sections were incubated for 72 hours with primary antibodies directly labelled with fluorophores diluted in 1% BSA with 0.1% Triton X-100 in PBS. After counterstaining with DAPI, sections were mounted on Superfrost+ slides using a frame (BioRad, Frame-Seal™ Slide Chambers, #SLF1201) and FluoromountG (Bioconcept, 0100-01).

#### H&E staining

Haematoxylin-Eosin (H&E) was performed using standard histological procedure to assess general morphology.

#### Confocal imaging and image analysis

Fixed infected and control LoCs were imaged using a Leica SP8 confocal microscope with a white light laser and 25x water immersion objective (NA = 0.95, Leica), with standard settings across chips labelled the same way. For chips processed via RNAscope, the excitation and emission windows were carefully chosen to minimize overlap of signal. Z-stacks were subsequently deconvolved using the Huygens Deconvolution Software (Scientific Volume Imaging). Imaris 9.9 (Bitplane) was used to analyse the intensity of immunofluorescence within the nucleus for Figures 2 and S3, nuclear volumes of infected cells in Figure 2, the bacterial volumes within lung tissue slices in Figures 3 and S2, the intensity of spots for RNAscope analysis in Figures 2 and S2, and the characterization of cords vs. clumps in axenic culture, LoC, and mouse models in Figures 1 and S1. Imaris was also used for rendering 3D images. ImageJ was used to analyse the size of Mtb microcolonies in infected macrophages in Figure 2 and for regrowth after antibiotic treatment in Figures 6 and S6.

#### Serial Block Scanning Electron Microscopy (SBSEM)

For SBSEM of infected ATs, a confluent layer of cells grown in 35 mm cell culture dishes with an underlying grid (Ibidi) were infected with Mtb. Between 3 and 5 dpi, they were fixed in 2% glutaraldehyde in 0.1M sodium cacodylate buffer at pH 7.4 for 1 hour. They were then rapidly screened via optical microscopy to identify suitable sites of cord formation, using a 20x air objective on a Nikon Ti-2 microscope. The samples were then postfixed in potassium ferrocyanide (1.5%) and osmium (2%) and stained with thiocarbohydrazide (1%) followed by osmium tetroxide (2%) alone. They were finally stained overnight in uranyl acetate (1%) and washed in distilled water at 50°C before staining with lead aspartate (pH 5.5) at the same temperature. The pieces were then dehydrated in increasing concentrations of alcohol and then embedded in Durcupan resin and hardened at 65°C for 24 hours. Once hardened, the regions for EM imaging were selected by matching their image with those taken prior to resin embedding. This region was trimmed from the rest of the resin and attached, with conductive glue, to an aluminium holder, then placed inside the scanning electron microscope (Merlin, Zeiss NTS) integrated with an in-chamber ultramicrotome device (3View, Gatan). Serial slices, 50 nm part, were cut from the block face, through the entire cell layer. Images were collected using a beam energy of 1.5 kV and 300 pA of current by collecting the backscatter signal. Images had a pixel size of 7.5 nm.

#### RNAscope assay

Infected chips were fixed for 2 hours at room temperature by immersing completely in a freshly diluted solution of 4% PFA in PBS and subsequently dehydrated in a sequence of washes with 50% EtOH, 70% EtOH, and 100% EtOH. Uninfected LoCs were maintained at an ALI for the same duration as the infected chips, to serve as matched controls, and then fixed and dehydrated similarly. The chips were then stored in 100% EtOH at -20°C until processed for RNAscope at the Histology Core Facility at EPFL. RNAscope Multiplex Fluorescent V2 assay (Bio-techne, Cat. No. 323110) was performed according to manufacturer’s protocol directly on chips. They were hybridized with a combination of the following probes Hs-*IL1B* (Bio-techne, Cat. 310361), Hs-IL6-C2 (Bio-techne, Cat. 310371-C2) Hs-IFNB1-C4 (Bio-techne, Cat. 417071-C4) simultaneously at 40°C for 2 hours. The different channels were revealed with TSA Opal570 (Akoya Biosciences, Cat. No. FP1488001KT), TSA Opal650 (Akoya Biosciences, Cat. No. FP1496001KT) and TSA Opal690 (Akoya Biosciences, Cat. No. FP1497001KT). Cells were counterstained with DAPI and mounted with Prolong Gold Antifade Mountant (Thermo Fisher, P36930).

#### LoC effluent immunoblot analysis

ALI medium flowing through the vascular channel of infected LoCs was collected over 4 dpi in sterile, hermetically sealed tubes. The effluent was filtered using a 0.2 μm pore size filter to eliminate any potential bacterial contamination and concentrated to 1 mL using Amicon ultracentrifugal filters with a 3 kDa cut-off (Millipore). Effluent collected from uninfected control LoCs were processed similarly. Immunoblot analysis for 23 human cytokines were performed using a 23-target Human Cytokine Antibody Array (Abcam, ab133996) as per manufacturer’s instructions. Briefly, array membranes were blocked at room temperature for 2 hours following which concentrated effluent samples were spotted on the printed side of the membrane and incubated for binding overnight at 4°C. Sample-bound membranes were washed extensively at room temperature with the wash buffers provided and then incubated with biotin-conjugated anti-cytokines overnight at 4°C. These membranes were washed to remove unbound anti-cytokines and incubated with HRP-conjugated streptavidin overnight at 4°C. At the end of the protocol, each membrane was washed and incubated with Detection Buffers for 2 minutes at room temperature. The array membranes were read using a Molecular Image ChemiDoc XRS+ System (BioRad).

#### RNA isolation

For RNA isolation from Mtb, 10 mL mid-logarithmic phase cultures were centrifuged at 5000 g for 5 minutes at 4°C, the pellet re-suspended in 800 μL of TRIzol Reagent (Thermo Fisher) and transferred to a 2 mL screw cap tube containing zirconia beads (BioSpec Products). Bacteria were disrupted by three 30 second cycles of bead-beating with rest on ice after every cycle. The lysate was transferred to a new tube, mixed with 200 μL of chloroform and centrifuged for 15 minutes at 12000 g at 4°C. The top aqueous phase was collected and mixed with an equal volume of isopropanol. The RNA was precipitated by centrifugation and purified.

For RNA isolation from infected macrophages in well plates, 800 μL of TRIzol Reagent was added to samples per well and collected by mechanical scraping. The cell lysate was mixed with 200 μL chloroform and centrifuged at 12000 g for 15 minutes at 4°C. The top aqueous phase was collected and mixed with an equal volume of isopropanol and brought out of the BSL-3 for RNA purification.

For collecting Mtb RNA from infected LoCs, 200 μL TRIzol reagent was added to both apical and vascular channels and samples were pooled from three different LoCs. In each case, the sample in TRIzol was transferred to a 2 mL screw cap tube containing zirconia beads (BioSpec Products). Cells were disrupted by three 30 second cycles of bead-beating (Precellys) with rest on ice after every cycle. The lysate was transferred to a new tube, mixed with 200 μL of chloroform and centrifuged for 15 minutes at 12000 g at 4°C. The top aqueous phase was collected and mixed with an equal volume of isopropanol. The RNA was precipitated by centrifugation.

For collecting host RNA from infected mouse lungs, ∼ 30 mg of tissue was immersed in 1 mL TRIzol Reagent and transferred to a screw cap tube with zirconia beads (diameter = 1.4 mm) for bead beating (Precellys) as described above. The lysate was transferred to a new tube, mixed with 200 μL of chloroform and centrifuged at 12000 g for 15 minutes at 4°C. The aqueous phase was collected and mixed with an equal volume of ice-cold isopropanol. The RNA was precipitated by centrifugation and washed with 70% ethanol. Subsequent processing of RNA for all TRIzol treated samples was carried out using the RNeasy Mini Kit for samples from mouse lungs or the RNeasy Micro Kit for in vitro samples, with on-column DNAse treatment for gDNA elimination steps as per manufacturer’s instructions.

For RNA isolation from eukaryotic cells in uninfected LoCs, samples were collected from apical and vascular channels separately in approximately 350 μL of the RLT Plus buffer of the RNeasy Micro Plus Kit and RNA was purified as per the manufacturer’s instructions.

#### Quantitative real-time PCR (qRT-PCR)

cDNA was generated using the SuperScript IV First-Strand Synthesis System with random hexamers (Invitrogen), and subsequently stored at -20°C. Specific primers used in this study are listed in the key resources table and were obtained from a commercial supplier (Microsynth, Switzerland). qRT-PCR reactions were prepared with SYBRGreen PCR Master Mix (Applied Biosystems) with 1 μM primers, and 1 or 2 μL cDNA. Reactions were run as absolute quantification on the ABI PRISM7900HT Sequence Detection System (Applied Biosystems) or QuantStudio 7 system (Applied Biosystems). Amplicon specificity was confirmed by melting-curve analysis.

#### RNA sequencing and data analysis

Libraries were prepared from ribodepleted RNA isolated from lung tissue taken from Mtb-infected mice and sequenced using the NEBNext® Ultra™ II RNA Library Prep Kit for Illumina® (New England Biolabs) as per the manufacturer’s instructions. A mean of 51.9 million 60 base-pairs paired-end reads were obtained among the 9 samples (39.2 – 69.3 million reads). The quality of sequenced Fastq files were analyzed using FastQC (version 0.11.9) and reads were mapped on the ensemble C3HeB/FeJ mouse reference genome (version 1.108) using STAR (v2.7.10b).^70^ The uniquely mapped reads were between 78.3%-91.6%. Reads count per gene was performed by STAR using option --*quantMode GeneCounts*.

Gene expression quantification was performed using R package edgeR.^71^ Genes below a mean of 10 reads count among the 9 samples were filtered out. Libraries normalization was performed using the TMM method and gene expression quantified as count per million (CPM).

Mouse lung module enrichment was performed using previously annotated modules.^72^ Module enrichment was calculated using the R package QuSAGE^73^ for the 3 following contrasts: Δ*pcaA* versus uninfected control; WT versus uninfected control; and Δ*pcaA* versus WT using log_2_CPM values. We used 2^17^ data-points for sampling the convoluted t-distribution and compute associated p-values and FDR adjusted p-values. Modules were finally filtered for significant (FDR<0.05) enrichment in any comparison investigated.

#### Gene Set Enrichment Analysis

Gene Set Enrichment Analysis was performed using GSEA software v4.3.2.^74^^,^^75^ The analysis was performed for 1000 permutations using the Gene Set “m8.all.v2023.1.Mm.symbols.gmt” and Permutation Type = Genotype.

#### Mtb protein extraction and Western blot

Mtb strains were inoculated in 10 mL cultures in Sauton’s medium supplemented with 0.05% Tween 80 at a starting OD_600_ of 0.1 and incubated at 37°C with shaking. After 4 days, the cells were harvested by centrifugation, washed with PBS, and resuspended in 10 mL of Sauton’s medium without Tween 80 and incubated again at 37°C with shaking for 4 days. Cultures were centrifuged and the pellets were collected. The pellets were resuspended in TBS buffer (20 mM Tris and 150 mM NaCl) supplemented with EDTA free protease inhibitor (Roche) and disrupted by bead-beating in four cycles of 30 seconds each using zirconia beads (BioSpec Products) and rest on ice during gaps to obtain bacterial lysates. The bacterial lysates were filtered through a 0.2 μm pore size filter and brought out of the BSL-3 facility. Protein samples were resolved using NuPAGE 4% to 12% bis-tris pre-cast gels (Invitrogen) under reducing conditions. Proteins were transferred from the gel to a nitrocellulose membrane using the iBlot Dry Blotting System (Invitrogen), according to manufacturer’s instructions. Membranes were blocked for 1 hour at room temperature with TBS supplemented with 3% BSA fraction V and then incubated overnight with the primary antibody diluted in TNT buffer

(TBS with 0.1% Tween-20) supplemented with 2% BSA fraction V at 4°C. Membranes were washed with TNT buffer, incubated with the secondary antibody in TNT supplemented with 2% BSA for 30 minutes at room temperature, washed with TNT buffer and developed using the Chemiluminescent Peroxidase Substrate (Sigma) on a Molecular Image ChemiDoc XRS+ System (BioRad). The following reagents were obtained through BEI Resources, NIAID, NIH: Monoclonal Anti-*Mycobacterium tuberculosis* GroEL2 (Gene Rv0440), Clone IT-56 (CBA1) (produced *in vitro*), NR-13655, Polyclonal Anti-*Mycobacterium tuberculosis* CFP10 (Gene Rv3874) (antiserum, Rabbit), NR-13801. A polyclonal secondary antibody conjugated with HRP was obtained from Dako, Denmark.

#### Langmuir monolayer measurements on Mtb lipids

A Langmuir-Blodgett balance with a continuous closed Teflon ribbon barrier (maximum area = 184 cm^2^, minimum area = 58 cm^2^; NIMA Technologies, Coventry, UK) was used to form monolayers of the surface lipids extractions. Langmuir monolayers were prepared by dropwise spreading the lipid extraction at the air-liquid interface, until the initial surface pressures were 1 mN/m (typically 15-30 μL were added). After allowing organic solvent evaporation for 10 min, the surface pressure vs. area isotherms were obtained by compressing at a barrier speed of 25 cm^2^/min, while constantly measuring the surface pressure by a sensor. The subphase consisted of double-distilled water maintained at 20, 25, 30 or 37 ± 1 °C, as appropriate.

#### Analysis of Langmuir monolayer measurements

##### The mycomembrane as a Langmuir monolayer

In comparison with WT, the levels of alpha and methoxy mycolic acids are depleted in the Δ*pcaA*, and the levels of keto mycolic acids are enriched in the Δ*pcaA*.^12^ Our hypothesis is that the hydrophobic interactions between lipids outer leaflet of the mycomembrane are responsible for the adhesion of Mtb bacteria to each other. We model the mycomembrane as a thin layer of free lipids, akin to a Langmuir monolayer^76^ - a thin 2D film of lipids on the surface of water.

A Langmuir-Blodgett apparatus^77^ probes the compressive properties of a Langmuir monolayer by measuring the change in surface tension γ as the monolayer is progressively compressed.

The surface pressure Π is related to the surface tension γ by:

(Equation 1) $$\Pi =\gamma_{0}\;–\;\gamma$$

where $\gamma_{0}=72$mN/m, the surface tension of water.

As the lipids in the Langmuir monolayer are pushed together, they exclude water molecules, which reduces γ and increases Π. This technique is typically used to study individual lipids; if used to characterize lipid mixtures, then the properties of the individual lipids are characterized first, and the lipids added at defined concentrations to generate a well-defined lipid mixture. Thus, an estimate of the packing density or the area per molecule is available throughout the compression cycle.

In this study, we measure isotherms of the entire surface Mtb lipids from WT or Δ*pcaA* to directly assess the effect of compression on surface lipid compositions as close to that in the mycomembrane composition as possible. The disadvantage of this unbiased approach is the inability to determine the specific composition of the different lipids and their precise concentration in the mixture. Starting lipid concentrations were chosen so that they would generate a measurable surface pressure based on the area compression achievable with the Langmuir trough used.

Lipid compressibility $C_{s}$ (the inverse of the compressional modulus) is defined as:

(Equation 2) $$C_{s}=-\frac{1}{A}\left(\frac{\partial A}{\partial \Pi }\right)$$

In the absence of quantification of the area per molecule, we are restricted to calculating dimensionless quantities using the relative area change due to compression. We therefore consider a normalized compressibility $C_{s0}$, which is normalized as follows:

(Equation 3) $$C_{s0}=\frac{C_{s}}{C_{s}\left(relative\;area=1\right)}$$

The Π−A isotherms for individual mycolic acids suggests that keto mycolic acids have the most rigid conformations.^48^^,^^49^ In MD simulations,^78^ keto mycolic acids in the gaseous phase adopt a **W** conformation which is maintained as the mycolic acids are compressed, whereas alpha and methoxy mycolic acids can be compressed into Π-shaped conformations that pack more efficiently.

The Π−A isotherm for WT lipids is relatively flat at intermediate compression values (Figure 4F), unlike for Δ*pcaA* lipids. This indicates the compressible nature of WT lipids versus Δ*pcaA* which is reflected in the sharp spike in $C_{s0}$ vs. relative area and Π (Figures 4F and S5D)

The relatively flat “shoulder” in the WT Π−A isotherm is indicative of a first order phase transition. At the given surface pressure (ca. 8 mN/m for the WT) where the shoulder occurs, the molecules are in a wide range of conformations - some compressed into **Π** configurations and others not. This is analogous to the melting of ice into water, or the boiling of water into steam. The energy stored in the changed conformations of the lipid molecules is termed latent heat L.

We now consider the formation of an Mtb cord. As bacteria grow and start to push against each other, the surface layer of lipids is likely compressed, analogous to the Langmuir monolayer (Figure 4E). Just as in the monolayer, at some point the lipids cannot be compressed further and are released to the surroundings (probably as vesicles). This releases the tension and might be accompanied by some slippage and reorientation between adjacent bacteria. In the agent-based simulations, we incorporate this as the collapse energy $E_{collapse}$threshold.

##### Estimation of energy storage

Based on the isotherms we argue the following:

(1) the work done in compressing a monolayer of Δ*pcaA* lipids ($W_{\Delta pcaA,\text{tot}}$) is the area under the curve of the Π–A isotherm. This is reversible work and is calculated via straightforward numerical integration as $W_{\Delta pcaA,rev}=-4.2152$ units.
(2) the work done in compressing a monolayer of WT lipids ($W_{WT,\text{tot}}$) is the sum of the area under the curve of the Π–A isotherm and the latent heat L. The first component, i.e., the reversible work is also calculated via numerical integration to provide $W_{\text{WT},\text{rev}}=-4.2389$ units.

To obtain an estimate for L we apply the 2D Clausius-Clapeyron relation from classical thermodynamics:

(Equation 4) $$\frac{dP}{dT}=\frac{L}{T\Delta A}$$

where P is the pressure at which the two phases co-exist and ΔA is the width of the shoulder.

The Π–A isotherms for WT lipids at four temperatures (20°C, 25°C, 30°C, 37°C) are shown in Figure S5E.

The first-order transition is not evident in the isotherms at 20°C and 25°C. This is not entirely unexpected, as the compressive properties of lipids are strongly temperature-dependent. Applying Equation 4 at 30 °C and 37 °C we get two estimates for L. These are:

$$L_{37}=-14.8433\;\text{units}\;\text{and}$$

$$L_{30}=-7.7498\;\text{units.}$$

Intuitively, these are likely to be upper and lower bound estimates. If we consider a value for L as the average of $L_{37}$ and $L_{30}$ that gives us the following final values:

$$W_{WT,tot}=W_{WT,rev}+L=-15.5355\;\text{units}\;\text{and}$$

$$W_{\Delta pcaA,tot}=W_{\Delta pcaA,rev}=-4.2152\;\text{units}$$

Thus, we conclude that WT Mtb lipids can store ca. 4-fold higher energy than those from Δ*pcaA*.

#### Agent-based simulations of Mtb growth

##### Overview

We model the mechanical and dynamical features of a TB filament through a minimal, agent-based model of colony formation and mechanics.^51^^,^^79^ Such models have been shown to reproduce several aspects of biofilm organization and dynamics, such as the emergence of ordered, chain-like subdomains^80^^,^^81^^,^^82^ and their response to internal and external fluxes.^51^^,^^83^ Here, we work with rod-shaped cells in 2D which interact through contact forces and contact torques. Cell growth is responsible for pressure within the colony.^80^ Additionally, we implement a threshold cell-cell adhesion force within the colony, whereby touching cells can only lose contact with each other if their separation forces exceed a critical collapse energy $E_{collapse}$.^82^ Our model demonstrates that a high $E_{collapse}$ allows for the emergence of long, locally-aligned filamentous structures, which we call cords. These cords have structural integrity and can sustain external forces while maintaining their cord architecture.

##### Model description

The TB filament is modelled as a collection of rod-shaped cells which grow, divide, and interact mechanically in 2D. To define the shape and position of a cell, we must specify its center, orientation, length, and radius. In our model, the center, orientation, and length can vary in time and from cell to cell, whereas the cell radius is constant. We let the center of the i’th cell be at $x_{i}\left(t\right)$, its orientation be $\theta_{i}\left(t\right)$ and its length be $b_{i}\left(t\right)$. The cells are symmetric, so the angle $\theta_{i}$ is only defined up to π. The cell radius is given by h for all cells and is time-independent. In addition to these geometrical parameters, each cell also has an age $a_{i}\left(t\right)$ and an age-limit, $A_{i}$, such that cell division occurs when $a_{i}$ reaches $A_{i}$.

The cell shapes can be thought of as thickened line segments. More precisely, we can define the 2D region occupied by the i’th cell at time t, $X_{i}\left(t\right)\subset R^{2}$, in terms of the quantities $\left(x_{i},\theta_{i},b_{i},h\right)$ (where we have suppressed the time dependence of $x_{i},\theta_{i},b_{i}$ for notational convenience). Let $n_{\theta_{i}}$ be a unit vector in the $\theta_{i}$ direction, and define the points $p_{i}^{1}$ and $p_{i}^{2}$ as follows:

$$\begin{array}{rr} p_{i}^{1} & =x_{i}-\frac{b_{i}}{2}n_{\theta_{i}}\\ p_{i}^{2} & =x_{i}+\frac{b_{i}}{2}n_{\theta_{i}} \end{array}$$

Let $L_{i}$ be the line segment from $p_{i}^{1}$ to $p_{i}^{2}$, so that the midpoint of $L_{i}$ is $x_{i}$. The cell is then defined to be the rod-shaped region consisting of points at most distance h from the line segment $L_{i}$.

(Equation 5) $$X_{i}\left(t\right)=\left\{x:\left|x-y_{i}\right|\leq h,y_{i}\in L_{i}\right\}$$

Since $x_{i}\left(t\right)$ is the midpoint of the line segment $L_{i}$, the i’th cell is therefore centered at $x_{i}\left(t\right)$. Each cell is a rod of length $b_{i}\left(t\right)$ with radius h. The boundary of the i’th cell, $\partial X_{i}$, is the set of points at distance h from $L_{i}$.

(Equation 6) $$\partial X_{i}\left(t\right)=\left\{x:\min_{y_{i}\in L_{i}}\left|x-y_{i}\right|=h\right\}$$

##### Dynamics

As described above, the i’th cell is defined by the quantities $\left(x_{i}\left(t\right),\theta_{i}\left(t\right),b_{i}\left(t\right),a_{i}\left(t\right),A_{i},h\right)$. The dynamics of $x_{i}$ and $\theta_{i}$ arise from contact forces and torques, and the dynamics of $b_{i}$ are due to an imposed growth law. Additionally, cell division occurs stochastically as the colony evolves, and depends upon cell age $a_{i}\left(t\right)$, and age limit $A_{i}$. We construct these equations in more detail below.

##### Growth law

The length of each cell increases linearly with time until cell division. The cell length $b_{i}$ satisfies:

(Equation 7) $${\dot{b}}_{i}=\alpha_{g}$$

where $\alpha_{g}$ is a constant growth rate.

##### Cell division

Cells divide in our simulation when their ages reach a particular limit. This age limit, $A_{i}$, is chosen randomly at birth for each cell as follows:

(Equation 8) $$A_{i}^{\prime }∼N\left(\mu_{a},\sigma_{a}^{2}\right),A_{i}=\text{Relu}\left(A_{i}^{\prime }\right)$$

Here, $A_{i}^{\prime }$ is normally distributed with mean $\mu_{a}$ and standard deviation chosen to be $\sigma_{a}=\mu_{a}/5$. We note that $A_{i}^{\prime }$ is positive with high probability:

$$P\left(A_{i}^{\prime }<0\right)<10^{-6}$$

so the distribution of $A_{i}$ is not significantly different from that of $A_{i}^{\prime }$.

Cell division is governed both by $A_{i}$ and the parameter $a_{i}\left(t\right)$, which tracks the age of cell i. We therefore have that ${\dot{a}}_{i}=1$ after cell i is born and before cell i divides. When $a_{i}\left(t\right)$ reaches $A_{i}$, the cell divides into two new cells, with indices j and k. Suppose the division occurs at time T, so $a_{i}\left(T\right)=A_{i}$. The post-division values of the geometric parameters x,θ,b for both of the new cells are then given as follows:

(Equation 9) $$\begin{array}{c} \theta_{j}{|}_{T^{+}}=\theta_{k}{|}_{T^{+}}=\theta_{i}{|}_{T^{-}}\\ b_{j}{|}_{T^{+}}=b_{k}{|}_{T^{+}}={\frac{1}{2}b}_{i}{|}_{T^{-}}\\ x_{j}{|}_{T^{+}}=x_{i}{|}_{T^{-}}+\left(\frac{1}{4}b_{i}{|}_{T^{-}}+h\right)n_{\theta_{i}}\\ x_{k}{|}_{T^{+}}=x_{i}{|}_{T^{-}}-\left(\frac{1}{4}b_{i}{|}_{T^{-}}+h\right)n_{\theta_{i}} \end{array}$$

The new cells j and k begin aging from 0

$$a_{j}\left(T\right)=a_{k}\left(T\right)=0{\dot{a}}_{j}={\dot{a}}_{k}=1$$

until their age-limit is hit. As before, these age-limits $A_{j}$ and $A_{k}$ are chosen randomly as specified in Equation 8. The form of cell division described above essentially cuts the parent cell in half.

##### Contact interactions

Cells i and j can interact through short-range contact forces and torques. To describe these cell-cell interactions, consider the line segments $L_{i}$ and $L_{j}$ which determine the shape of cells i and j (Equation 5). Let $q_{ij}$ be the point on $L_{i}$ which is closest to $L_{j}$

$$q_{ij}=\min_{q\in L_{i}}d\left(q,L_{j}\right)$$

where $d\left(q,L_{j}\right)$ denotes the minimum distance between a point q and the line segment $L_{j}$. Let $r_{ij}$ be the shortest vector from $L_{i}$ to $L_{j}$

$$r_{ij}=q_{ji}-q_{ij},r_{ij}=\left|r_{ij}\right|,{\hat{r}}_{ij}=\frac{r_{ij}}{r_{ij}}$$

Since cells i and j have radius h, they are in contact if $r_{ij}\leq 2h$. We define $p_{ij}$ in terms of $r_{ij}$ as follows:

(Equation 10) $$p_{ij}=2h-r_{ij}$$

so that the collision criterion is equivalent to $p_{ij}\geq 0$. We model the contact forces on cells through a soft contact potential, V(p), which is 0 for p<0 and increases and stiffens nonlinearly for p>0

(Equation 11) $$\begin{array}{r} V\left(p\right)=\left\{ \begin{array}{ll} \frac{1}{2}K_{r}\left(p^{2}+50p^{4}\right) & \text{if}\;p>0\\ 0 & \text{if}\;p<0 \end{array}\right. \end{array}$$

where $K_{r}$ is a constant capturing the bulk elasticity of the cells. $K_{r}$ has dimensions of force, and p has dimensions of length, so V is an energy. The total contact force on the i’th cell then follows from differentiating V(p)

(Equation 12) $$F_{i}^{\text{con}}=-{\sum_{j}V}^{\prime }\left(p_{ij}\right){\hat{r}}_{ij}$$

Note that the contact force on the i’th cell is in the direction $-r_{ij}$, which points from cell j to cell i, as expected.

In addition, we assume that cells which are in contact must overcome an energy barrier, $E_{c}$, in order to separate. In other words, if cells i and j are in contact, so $p_{ij}>0$, then an energy threshold $E_{c}$ is required to reach the $p_{ij}<0$ state. We can write this in terms of a potential energy, $V_{\text{sep}}\left(p\right)$, which applies when cells are separating, i.e., when $\dot{p}<0$.

(Equation 13) $$\begin{array}{r} V_{\text{sep}}\left(p\right)=\left\{ \begin{array}{ll} \frac{1}{2}K_{r}\left(p^{2}+50p^{4}\right) & \text{if}\;p>0\\ E_{c} & \text{if}\;p<0 \end{array}\right. \end{array}$$

Note that $V^{\prime }\left(p\right)=V_{\text{sep}}^{\prime }\left(p\right)$ for p≠0, so both potentials give rise to the same forces away from p=0. This separation energy barrier at p=0 can be thought of as a threshold adhesion force, keeping two cells in contact unless separation forces become sufficiently large. To write down the total force on cell i, we observe that the potential energy of the interaction between cells i and j is $V\left(p_{ij}\right)$ if ${\dot{p}}_{ij}>0$ (Equation 11) and $V_{\text{sep}}\left(p_{ij}\right)$ if ${\dot{p}}_{ij}<0$ (Equation 13). The total force on cell i is therefore:

$$F_{i}^{\text{tot}}=\sum_{j}\left(-V^{\prime }\left(p_{ij}\right)\;\Theta \left({\dot{p}}_{ij}\right)-V_{\text{sep}}^{\prime }\left(p_{ij}\right)\;\Theta \left(-{\dot{p}}_{ij}\right)\right){\hat{r}}_{ij}$$

where Θ is the Heaviside step function, satisfying Θ(p)=1 if p>0 and Θ(p)=0 if p<0. To simplify the above expression for $F^{\text{tot}}$, we use the fact that $V^{\prime }\left(p\right)=V_{\text{sep}}^{\prime }\left(p\right)$ for p≠0, and that at p=0 we have:

$$V_{\text{sep}}^{\prime }\left(p\right){|}_{p=0}=-E_{c}\delta \left(p\right){|}_{p=0}$$

where δ(p) is the delta function. We can therefore write $F^{\text{tot}}$ as

(Equation 14) $$F_{i}^{\text{tot}}=\sum_{j}\left(-V^{\prime }\left(p_{ij}\right)+E_{c}\delta \left(p_{ij}\right)\;\Theta \left(-{\dot{p}}_{ij}\right)\right){\hat{r}}_{ij}$$

The first term in the above expression is the contact force (Equation 12). The second term is an adhesion force which acts when cells are on the point of separating, i.e., when $p_{ij}=0$ and ${\dot{p}}_{ij}<0$

$$F_{i}^{\text{adh}}=E_{c}\sum_{j}{\hat{r}}_{ij}\;\delta \left(p_{ij}\right)\Theta \left(-{\dot{p}}_{ij}\right)$$

We therefore have

$$F_{i}^{\text{tot}}=F_{i}^{\text{con}}+F_{i}^{\text{adh}}$$

Finally, each cell experiences a torque arising from interaction forces. The total torque on cell i is:

(Equation 15) $$\tau_{i}^{\text{tot}}=\sum_{j}\left(-V^{\prime }\left(p_{ij}\right)+E_{c}\delta \left(p_{ij}\right)\;\Theta \left(-{\dot{p}}_{ij}\right)\right)\left(q_{ij}-x_{i}\right)\wedge {\hat{r}}_{ij}$$

##### Equations of motion

We assume overdamped dynamics, with translational and rotational friction parameters $\gamma_{T}$ and $\gamma_{R}$ respectively for each cell. Additionally, we include random forces $\xi_{i}^{T}\left(t\right)$, and torques $\xi_{i}^{R}\left(t\right)$, for each cell, leading to the following equations of motion:

(Equation 16) $$\begin{array}{rr} \gamma_{T}{\dot{x}}_{i} & =F_{i}^{\text{tot}}+\xi_{i}^{T}\\ \gamma_{R}{\dot{\theta }}_{i} & =\tau_{i}^{\text{tot}}+\xi_{i}^{R} \end{array}$$

Here $\xi_{i}^{T}$ and $\xi_{i}^{R}$ are independent white noise processes satisfying:

$$\left\langle {\left(\xi_{i}^{T}\right)}_{\mu }\left(t\right)\;{\left(\xi_{i}^{T}\right)}_{\nu }\left(t^{\prime }\right)\right\rangle =2D_{T}\;\delta_{\mu \nu }\;\delta \left(t-t^{\prime }\right),\left\langle \xi_{i}^{R}\left(t\right)\;\xi_{i}^{R}\left(t^{\prime }\right)\right\rangle =2D_{R}\;\delta \left(t-t^{\prime }\right)$$

where angle brackets denote expected value, the indices μ,ν take values 1,2, and $D_{T},D_{R}$ are constants setting the variance of the white noise processes. Over a time, interval Δt, the typical magnitudes of the random forces and torques are given by:

$$F^{\text{noise}}=\sqrt{\frac{2D_{T}}{\Delta t}},\tau^{\text{noise}}=\sqrt{\frac{2D_{R}}{\Delta t}}$$

##### Simulation parameters

The following table provides the parameter details for Figures 4 and 5 and Videos S3 and S4 in this study.

**Table:** 

Figure/Video	$K_{b}$	$E_{collapse}$	$F_{noise}$	$F_{ext}$	Dt	Replicates
4G, Video S3	0.1	0, 9	0.004	0	0.1	1
4H	0.1	0-9	0.0001-0.01	0	0.1	20
4I	0.1	0-9	0.0001-0.01	0	0.1	20
5A, Video S4	0.1	0, 9	0.0001	0, 0.5	0.1	1
5B	0.1	0-9	0.004	0 – 0.5	0.1	1
5C, 5D, 5E	0.1	0-9	0.001	0-1	0.1	25

The code is uploaded to Zenodo: 10.5281/zenodo.7740873 .

#### Determination of Minimum Inhibitory Concentration in axenic culture

To determine the minimum inhibitory concentration of an antibiotic, microplate Alamar Blue assay was performed in a 96 well flat bottom plate (Greiner Bio-One). Mtb strains grown to mid-exponential phase were diluted to OD_600_=0.0006 and incubated with serial dilutions of antibiotics in 7H9 medium at 37°C. After 5 days of incubation, 10 μL of Alamar Blue reagent (Thermo Fisher Scientific) was added to all wells, including only medium and no drug control wells, and the plate was incubated for another 24 hours at 37°C. At the end of the incubation, fluorescence readings were recorded in a TECAN microplate reader at 560 nm excitation wavelength and 590 nm emission wavelength with a 20 nm emission bandwidth at 100% gain. Percentage survival of bacteria was calculated by subtracting background for reagent from the readings of the medium only wells and normalized to uninhibited bacterial growth from the no drug control wells and analyzed using the Gompertz fit^84^ for MIC determination in GraphPad Prism.

#### Analysis of bedaquiline adsorption to the PDMS in the LoC model

The adsorption of antibiotic molecules to PDMS can impact the efficacy of antibiotic delivery to foci of infection in the LoC model. Grant et al.^56^ generated a finite element simulation drug delivery in the same architecture as the LoC model used in the present study. Loss of antibiotic occurs through adsorption to the PDMS walls of the vascular channel and subsequent diffusion within PDMS. This former is quantified by the partition coefficient $P_{PDMS}$ in PDMS vs. media in the vascular channel, defined as:

(Equation 17) $$P_{PDMS}=\frac{c_{PDMS}}{c_{media}}$$

and the latter by $D_{PDMS}$ the diffusion coefficient of the drug in PDMS.

Based on the simulations, the quantity of drug flowing through the vascular channel is sensitive to both $P_{PDMS}$ and $D_{PDMS}$. $D_{PDMS}$ cannot be quantified without fluorescent analogues of BDQ and INH. However, as BDQ and INH are of roughly the same molecular weight as the drug Amodiaquine, we can assume they have roughly comparable $D_{PDMS}$ values as that calculated for Amodiaquine by Grant et al.^56^

Estimates for $P_{PDMS}$ can be obtained from data on partition coefficients in octanol from the DrugBank database.^85^ INH has a high solubility in water and a low partition coefficient in octanol (logP=−0.71,P=0.194); the simulations predict a rapid saturation of INH within the vascular channel, at concentrations that correspond to >90 % of the input. BDQ is highly lipophilic^85^ with a very high partition coefficient in octanol ($\log\;P=6.37,P=2\times 10^{6}$). This is several orders of magnitude above the highest $P_{PDMS}$ values simulated by Grant et al. The simulations would suggest that BDQ concentration in the vascular channel would only be a very small fraction of the input concentration, that it would take a very long period of time to reach a steady state, and that the highest BDQ concentrations would be found nearest the inlet of the LoC and diminish as distance from the inlet increases (schematic in Figure S6N).

This last prediction can be tested with measurements of Mtb regrowth. BDQ is bactericidal; regrowth rates would be expected to correlate negatively with local BDQ concentrations. However, no spatial trend is observed for regrowth rates (Figure S6N). This suggests that the apical BDQ levels may be higher than predicted by the model of Grant et al. This could be because of several reasons. First, BDQ adsorption to the PDMS walls of the LoC may be reduced due to the pre-coating of the walls of the channel with extracellular matrix proteins and serum proteins from the cell culture media. Second, BDQ is also reported to strongly bind to proteins in human serum,^55^ a similar mechanism may also operate in the LoC model. Third, BDQ uptake by cells in the LoC model and intracellular sequestration could increase the bioavailability of BDQ to intracellular Mtb. A complete dissection of these different mechanisms is beyond the scope of this study.

### Quantification and statistical analysis

Statistical analyses are described in each figure legend. For experiments combining several groups, one-way ANOVA with the Dunn’s multiple comparison test was used (Figures 2A–2D, 2I–2L, 6A, 6B, S1F, S1Q, S2, S3B, S3C, S3I, S3J, S4D, S4E, S6D, S6F–S6H, S6K, and S6L). For comparing a change in mean for the experimental test group, a one sample t test with mean set at zero was used (Figures 6H–6K, S6N, and S6O). For comparisons between two experimental groups, the two-tailed Mann-Whitney (Figures 1G, 1H, 1M, 2F, 2M, 3B, 3C, 3E, S1D, S1G–S1I, S1R, S3F, S4C, and S6M) or Welch’s test was used (Figure S4G). Statistical significance was determined using Prism v.9.5.0 software (GraphPad). P > 0.05 was considered non-significant. For RNAscope analysis, fields of view from a given LoC were considered as biological replicates, and the number of LoCs corresponds to the number of times the experiment was repeated. All data were obtained from at least *n* = 2 independent experiments and quantifications for histological analysis were performed by *n* = 2 independent investigators.

## Data Availability

• Bulk-RNA seq data have been deposited at GEO and are publicly available as of the date of publication. Accession numbers are listed in the key resources table. Microscopy data for panels in the Figures have been uploaded to Zenodo and are publicly available as of the date of publication. The DOI is listed in the key resources table. • All original code has been deposited at Zenodo and is publicly available as of the date of publication. DOIs are listed in the key resources table. • Any additional information required to reanalyze the data reported in this paper is available from the lead contact upon request.
